# Waist circumference, waist‐to‐height ratio and BMI percentiles in children aged 5 to 19 years in India: A population‐based study

**DOI:** 10.1002/osp4.493

**Published:** 2021-03-23

**Authors:** Avina Sarna, Akash Porwal, Rajib Acharya, Sana Ashraf, Sowmya Ramesh, Nizamuddin Khan, Sikha Sinha, Harshpal Singh Sachdev

**Affiliations:** ^1^ Population Council Zone 5A Ground Floor India Habitat Centre New Delhi India; ^2^ Sitaram Bhartia Hospital New Delhi India; ^3^ Pediatrics and Clinical Epidemiology Sitaram Bhartia Institute of Science and Research New Delhi India

**Keywords:** BMI, children, India, obesity, waist circumference, waist‐to‐height ratio

## Abstract

**Objective:**

Nationally representative percentiles for waist circumference (WC), waist‐to‐height‐ratio (WHtR), and body mass index (BMI) are not available for children and adolescents in India.

**Methods:**

Using LMS method, age‐ and gender‐specific reference growth charts were constructed for WC (*n* = 68,261), WHtR (*n* = 68,261), and BMI (*n* = 67,741) from children/adolescents aged 5–19 years who participated in a nationally representative survey. General obesity, indicating overall obesity, was defined as age–sex‐specific BMI *z*‐scores ≥ 95th percentile. Central obesity was defined in three ways: WC ≥ 90th percentile, WHtR ≥ 0.5, and both WC ≥ 90th percentile and WHtR ≥ 0.5.

**Findings:**

WC and BMI percentiles for boys and girls are lower than those previously reported from India and several other countries. The BMI percentiles are lower than the WHO 2007 reference population. The prevalence of general obesity using India specific BMI centiles was 2.9% (95% CI: 2.6–3.2). The prevalence of central obesity was 6.1% (95% CI: 5.7–6.6) using WC ≥ 90th percentile, 5.3% (95% CI: 5.0–5.7) using WHtR ≥ 0.5, and 3.6% using both criteria. Three‐fourth of children with general obesity also had central obesity based on WC ≥ 90th.

**Conclusions:**

Indian children are thinner than Caucasian and other Asian children, and the global WHO reference population. Using India specific reference, the prevalence of central obesity is higher than general obesity with a significant overlap between the two.

## 1 INTRODUCTION

The association of obesity with cardio‐metabolic risk is well documented.^1^ The increasing prevalence of childhood and adolescent obesity in India raises concerns about obesity persisting into adulthood with long‐term cardio‐metabolic consequences, such as hyperlipidemia, coronary heart disease, and diabetes.^2^ There are varying estimates of child and adolescent obesity in India. A systematic analysis conducted as part of the Global Burden of Disease study estimated that 2.3% of males and 2.5% of females aged under 20 years in India were obese.^3^ A recent systematic review of 52 small‐scale studies examining obesity among children and adolescents conducted in 16 states of India, reports a combined prevalence estimate of obesity of 19.3% between 2010 and 13, up from 16.3% reported in 2001–2005.^4^ The review also highlights differentials in trend with a greater increase in prevalence reported from the northern and eastern parts of the country, in urban areas, and in the higher socio‐economic status. The increasing prevalence makes screening for obesity in children and adolescents critical for the early introduction of prevention initiatives.

The use of body mass index (BMI) to define overweight and obesity is well established, and the World Health Organization (WHO) provides BMI‐for‐age and sex percentiles defining overweight as BMI‐for‐age >+1SD (equivalent to BMI 25 kg/m^2^ at 19 years age) and obese as BMI‐for‐age >+2SD (equivalent to BMI 30 kg/m^2^ at 19 years age) drawn from a global reference population (WHO 2007).^5^ However, it has been suggested that BMI may not accurately assess adiposity, particularly central adiposity, as it measures body weight that includes lean muscle mass and does not distinguish between fat and fat‐free mass. BMI is a better measure of general adiposity.^6^ Waist circumference (WC), on the other hand, is considered a better marker of central adiposity,^6^, ^7^ and a good predictor of cardio‐metabolic risk; there are several expert recommendations to include WC measurement in routine clinical vital signs.^8^ Several studies have used a cutoff of WC ≥ 90th percentile to indicate obesity.^9^, ^10^, ^11^ There is also increasing interest in the use of Waist‐to‐Height Ratio (WHtR) to assess obesity among children and adolescents. A ratio of greater than 0.5 is considered indicative of obesity.^12^, ^13^ WHtR is more closely linked to childhood morbidity than BMI, and has been documented to be a critical indicator for children and adolescents.^13^ A recent evaluation of six indices: BMI, WC, WHtR, waist‐to‐hip ratio, log of sum of triceps, and subscapular skin fold thickness and bioimpedance‐based percent body fat, found WC and WHtR to be the most predictive indices to identify South Asian adults with cardio‐metabolic risk.^14^ Asians and Indians, in particular, appear to have higher body fat per unit of BMI than western populations, and thus, require population specific percentiles.^7^, ^15^ Indian children are prone to develop central obesity highlighting the need for early intervention.^16^ Over the last decade, several groups have applied the LMS model to create smoothed percentile charts for WC, and in some countries WHtR in children and adolescents from Malaysia,^10^ Pakistan,^9^ Poland,^11^ and Turkey^17^ among others. In India, most studies have created LMS percentile curves for WC and WHtR among children and adolescents from urban schools.^18^ Similarly, BMI percentiles have been created with children from urban, middle‐to‐upper socio‐economic strata.^19^, ^20^ India is a diverse country with 70% rural population and varied economic status and ethnicity. Presently, percentile curves for WC, WHtR, and BMI drawn from a national representative population of Indian children/adolescents are not available. Obtaining representative, normative information for WC, WHtR, and BMI is necessary for reliable identification and prevention of overweight and obesity and associated cardio‐metabolic risk factors among Indian children.

The LMS method was used to create age‐ and gender‐specific reference growth charts for WC, WHtR, and BMI in Indian children and adolescents aged 5–19 years using data from the nationally representative Comprehensive National Nutrition Survey (CNNS 2016–2018). Based on India‐specific percentiles, the prevalence of general and central obesity was determined, and socio‐demographic differentials examined with the objective of informing national policy and programmes, and to serve as a baseline for future comparisons.

## 2 METHODS

The CNNS was conducted under the aegis of the Ministry of Health and Family Welfare (MoH&FW) in collaboration with UNICEF and the Population Council. The CNNS was designed to provide nationally representative and comprehensive nutritional profiling of preschoolers (0–4 years), school‐age children (5–9 years) and adolescents (10–19 years), based on biological sample assessment and multiple anthropometric measures. This paper focuses on school‐age children and adolescents (5–19 years).

### 2.1 Study design and participants

The survey design and methodology are published elsewhere.^21^ Briefly, the CNNS used a multi‐stage, stratified, probability proportion to size cluster sampling design to select a nationally representative sample of households and individuals aged 0–19 years across all 29 states of India and the capital Delhi. Households with individual(s) between 0 and 19 years were randomly selected from rural and urban primary sampling units (PSU); children/adolescent members were classified into three strata (0–4, 5–9, and 10–19 years), and only one child/adolescent was selected from each stratum per household. The sample size was set at 122,100 (40,700 in each age group) from 2035 PSUs to provide national, state‐level, and rural‐urban estimates.^21^ Children/adolescents who had a chronic illness, physical deformity, mental illness or cognitive disability, or any ongoing current illness (fever, infection) were excluded from the survey. The survey collected socio‐demographic data: place of residence, wealth index, religion, caste, mother's education, safe water, and sanitation from questionnaires and anthropometry data.^21^

### 2.2 Study sample

**FIGURE 1:**
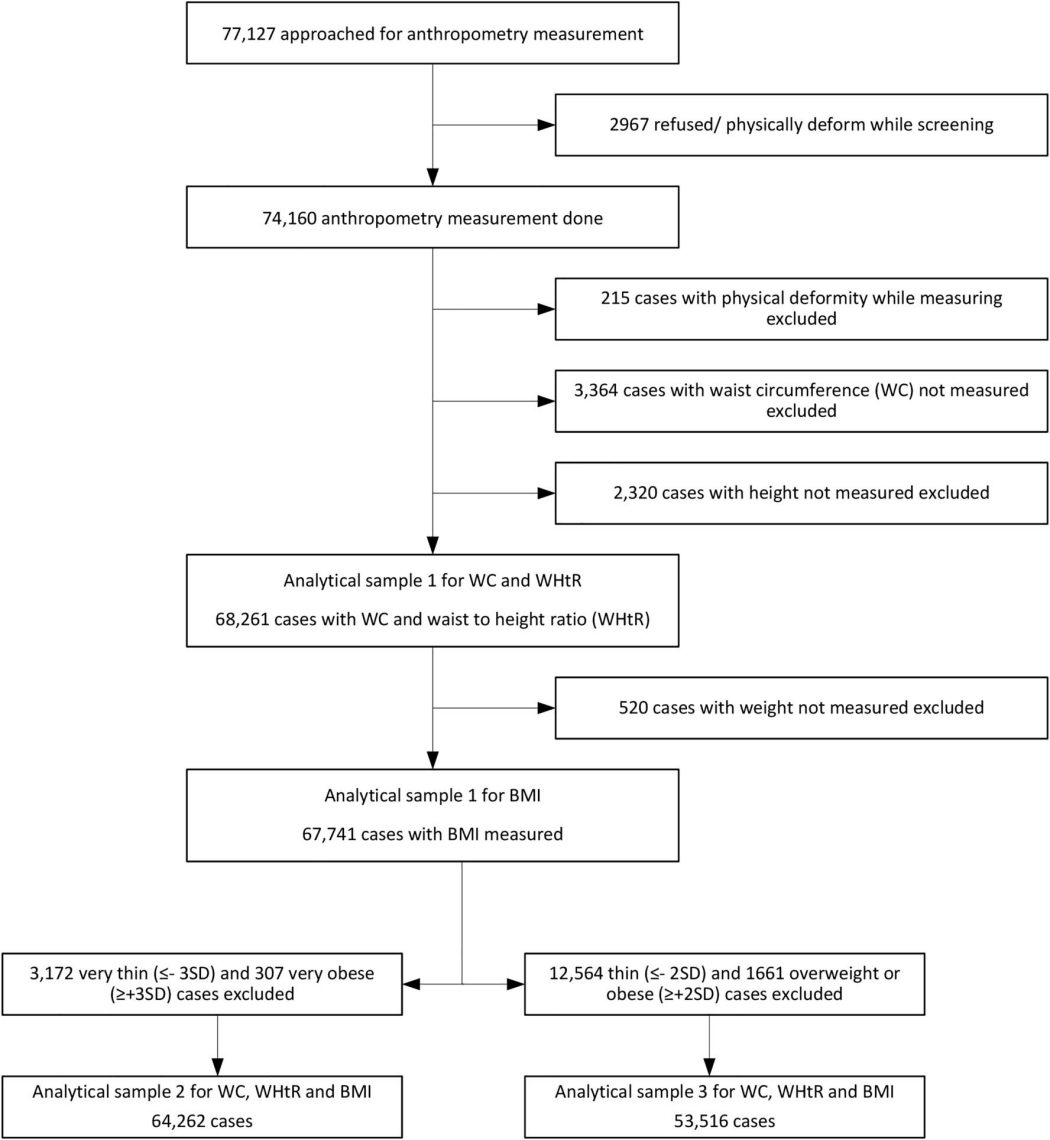
Flow chart for the analytical sample which included all participants with height, waist circumference, and BMI measurements

Children/adolescents aged 5–19 years were included in this analysis. Participants for whom data on height, weight, or WC was missing were excluded from this analysis (Figure 1). At the time of anthropometric measurement, a few participants were detected to have a physical deformity that was not evident at the time of recruitment into the survey (e.g., scoliosis, kyphosis, bow‐legs etc.); these participants were excluded from the analysis (*n* = 215). We created three analytical samples. Our first analytical sample included all the eligible participants. As a sensitivity analysis, we constructed two reference populations: (i) after excluding very thin (<−3SD) and very obese (>+3SD) participants (analytical sample 2) and (ii) after excluding thin (<−2SD) and obese (>+2SD) participants (analytical sample 3) based on age‐ and sex‐adjusted BMI *z*‐scores using the WHO 2007 growth reference chart (Figure 1).

Ethical approvals for the survey were obtained from the Ethics Committee of the Postgraduate Institute for Medical Education and Research in Chandigarh, India, and the Institutional Review Board of the Population Council in New York. Written informed consent was obtained from caregivers of children aged 5–10 years· For adolescents aged 11–17 years, written consent was obtained from their caregivers and written assent obtained from the adolescents· Adolescents aged 18–19 years provided their own consent. Publicly available open source data have been used for secondary analyses in this paper and did not require further approvals or consent.

### 2.3 Anthropometric measurements

The anthropometric parameters included were height, weight, and waist circumference. Trained female health workers collected all anthropometric data. Height was measured in centimeters on a SECA height board (to the nearest 0.1 cm); the mean of two readings was recorded. Weight was measured in kilograms (up to 0.01 kg) using a SECA portable digital weighing scale; only one reading was taken/recorded. Waist circumference was measured in centimeters (to the nearest 0.1 cm) at the midpoint between the lowest rib and the iliac crest in the mid‐axillary line at the end of normal expiration using a non‐elastic measuring tape.^22^ The mean of two readings was recorded. Rigorous quality monitoring was maintained including weekly calibration of the height board and daily calibration of the weighing scale and repeat measurements by quality monitors (CNNS report).^21^ For height measurement, the inter‐ and intra‐technical error of measurement (TEM) scores were within the global cutoffs of 0.95 and 0.69 cm.^23^ There are no global TEM cutoffs for WC.

### 2.4 Statistical methods

The LMS method was used to compute age‐ and sex‐specific percentiles for WC, WHtR, and BMI. WHtR was calculated as waist (centimeters)/height (centimeters), and BMI was calculated as weight (kgs)/height^2^ (meters). Each measurement was summarized by three smooth curves plotted against age representing the median (M), coefficient of variation (S) and skewness (L) of its distribution. The Box‐Cox‐Cole‐Green (BCCG) distribution with penalized spline smoothing was used to construct smoothed age‐sex specific percentile curves of WC, WHtR, and BMI for the three analytical samples.^24^ LMS values and percentiles were calculated using the general additive models for location, scale, and shape(GAMLSS) 4.3‐1 library under R3.1.2.^25^ Goodness of fit of the models was accessed by the Bayesian information criterion and by Q‐Q plots.^26^ Age‐adjusted *z*‐scores for WC, WHtR, and BMI were calculated by regressing WC, WHtR, and BMI, respectively, on age by sex and standardized residuals from each model represented age‐adjusted WC, WHtR, and BMI *z*‐scores. WC, WHtR, and BMI values at percentiles 5, 25, 50, 75, 85, 90, and 95 were obtained separately for boys and girls. R software version 3.6.1 was used for developing the percentiles. The 50th and 90th percentile curves for WC of the study population were compared with those of children/adolescents from selected countries (US[NHANES],^27^ Poland,^11^ Turkey,^17^ Malaysia,^10^ and Pakistan^9^), and a study by Khadlikar and colleagues^18^ for an Indian population that used the same measurement methods. Similarly, BMI 50th and 90th percentile curves were compared with India (Indian Academy of Pediatrics),^19^ Malaysia,^28^ Poland,^29^ Turkey^30^ and the WHO global reference population.^5^

For sensitivity analysis, data distribution of the reference populations in analytical samples 1: all eligible participants, sample 2: excluding >+3SD and <−3SD participants and sample 3: excluding >+2SD and <−2SD were compared. Age‐ and sex‐specific 50th and 90th percentiles for WC and 50th and 95th percentiles for BMI were developed with 95 percent confidence intervals (95% CI) using raw values of WC and BMI. 95% CIs were calculated using simple standard error and bootstrap method with 1000 repetitions. The 95% CI in analytical samples 1, 2, and 3 were similar for 90th percentile for WC and 95th percentile for BMI indicating no significant differences at values used to define obesity (Figure S3).

WHO defines obesity as age‐ and gender‐specific BMI *z*‐scores =>2SD (95th percentile) from percentiles; we used this cutoff based on newly constructed percentile reference charts to define general obesity.^5^ The National Health and Nutrition Examination Survey (NHANES) has proposed an age‐ and sex‐specific cutoff of ≥90th percentile of WC for identifying central obesity; this cutoff has been used by several studies.^1^ ^,^ ^10^ ^,^ ^11^ ^,^ ^31^ WHtR has also been used to define central obesity with a fixed cutoff of ≥0.5.^32^ Recently, studies have combined WC and WHtR to define central obesity, as age‐and sex specific WC percentile ≥ 90th and WHtR ≥ 0.5.^9^ Central obesity is reported based on all three indicators: WC ≥ 90th percentile, WHtR > 0.5, and both WC ≥ 90th percentile and WHtR > 0.5. Sex‐specific prevalences of central and general obesity, as defined above, are reported. Bivariate analyses used the ANOVA test to compare the mean *z*‐scores of outcome variables (WC, WHtR, BMI) across socio‐demographic variables; this was conducted in the weighted sample to account for differences in probabilities of selection across states and non‐response rates. These analyses were conducted using STATA version 16.0 (College Station, TX).

## 3 RESULTS

Between February 2016 and October 2018, anthropometry data were collected from 74,160 children aged 5–19 years of age (38,331 school‐age children [5–9 years] and 35,829 adolescents [10–19 years]). The first analytical sample comprised 68,261 and 67,741 subjects for WC and WHtR, and BMI, respectively (Figure 1). The second analytical sample for WC, WHtR, and BMI comprised 64,262 participants and the third analytical sample comprised 53,516 participants (Figure 1).

Relevant descriptive statistics are summarized in Table 1. The mean (SD) for age, weight, height, WC, and WHtR was 11.8 (4.1) years, 32.3 (13.5) kg, 137.9 (19.4) cm, 58.5 (8.7) cm, and 0.43 (0.04), respectively. The mean BMI was 16.2. Overall, 4.6% (3172/67,741) of the study population was very thin (BMI *z*‐score ≤ 3SD) and 0.1% (307/67,741) was very obese (BMI *z*‐score≥3SD); more boys were thin compared to girls (7.7% vs. 4.1%) (data not shown). Detailed age‐ and sex‐specific descriptive statistics are provided in Table S1.

**TABLE 1:** Characteristics of a population‐based sample of children and adolescents 5–19 years in India (2016–2018)

Characteristics	Boys		Girls		Total	
	*n*	Mean (SD)	*n*	Mean (SD)	*n*	Mean (SD)
Age (years)	35,449	11.7 (4.1)	32,812	11.9 (4.1)	68,261	11.8 (4.1)
Weight (kg)	35,390	33.0 (14.4)	32,766	32.1 (12.5)	68,156	32.3 (13.5)
Height (cm)	35,449	139.6 (20.9)	32,812	136.2 (17.5)	68,261	137.9 (19.4)
Waist circumference (cm)	35,449	59.0 (8.9)	32,812	58.0 (8.4)	68,261	58.5 (8.7)
Waist circumference height ratio	35,449	0.42 (0.04)	32,812	0.43 (0.04)	68,261	0.43 (0.04)
Body mass index	35,130	16.0 (2.9)	32,611	16.3 (3.2)	67,741	16.2 (3.1)

### 3.1 Percentiles

**FIGURE 2:**
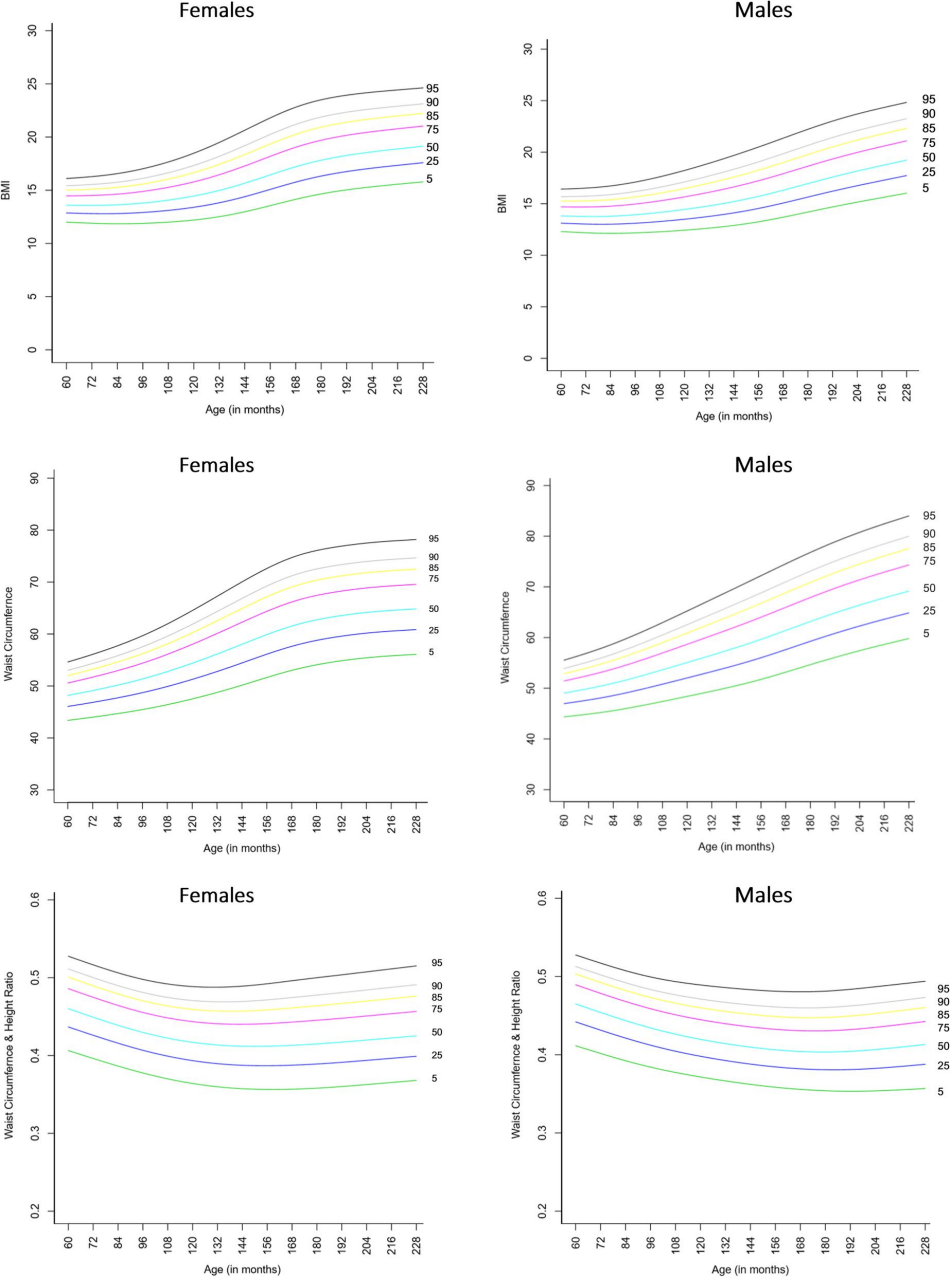
Smoothed waist circumference, waist to height ratio, and BMI percentiles curves for males and females aged 5–19 years in India (2016–2018)

WC and WHtR percentiles are presented in Figure 2. Corresponding percentile values and LMS parameters are presented in Table 2. WC increased with age in both boys and girls; there were marked sex differences in the shape of centile curves. Girls had lower WC values than boys at any age and percentile, and these differences increased with age. From around age 15 years the curve began to level off in girls, whereas the curve continued to rise for boys. There were no significant differences in the percentile curves and corresponding WC values with the “reference” population (sample 2 excluding the extremely thin (−3SD) and extremely obese (+3SD) [Table S2, Figure S1]) With regard to sample 3 (excluding thin (−2SD) and obese (+2SD) participants), there were no differences in the percentile curves and corresponding WC values at higher percentiles; however, at percentiles below the median significant differences were observed with higher centile values than sample 1 (Table S3, Figure S2).

**TABLE 2:** Smoothed percentile values of WC (cm), WHtR, and BMI for males and females aged 5–19 years in India (2016–2018)

Age (years)	Males										Females									
	L	M	S	P5	P25	P50	P75	P85	P90	P95	L	M	S	P5	P25	P50	P75	P85	P90	P95
WAIST CIRCUMFERENCE (WC) (*N* = 68,261)																				
5	−1.877	49.057	0.067	44.36	46.97	49.06	51.44	52.86	53.90	55.54	−1.538	48.191	0.069	43.39	46.06	48.19	50.58	52.00	53.02	54.62
6	−1.867	49.945	0.072	44.91	47.69	49.94	52.53	54.09	55.23	57.04	−1.540	49.123	0.073	44.00	46.85	49.12	51.70	53.23	54.34	56.09
7	−1.857	50.992	0.076	45.58	48.56	50.99	53.81	55.51	56.76	58.77	−1.541	50.160	0.077	44.68	47.72	50.16	52.95	54.62	55.82	57.74
8	−1.846	52.247	0.080	46.43	49.62	52.25	55.31	57.18	58.55	60.77	−1.542	51.355	0.081	45.47	48.72	51.36	54.39	56.22	57.55	59.67
9	−1.836	53.635	0.084	47.40	50.81	53.64	56.96	59.00	60.51	62.96	−1.543	52.766	0.086	46.41	49.90	52.77	56.09	58.10	59.57	61.94
10	−1.826	55.074	0.089	48.40	52.04	55.07	58.67	60.90	62.55	65.24	−1.545	54.381	0.091	47.51	51.27	54.38	58.02	60.24	61.87	64.51
11	−1.815	56.529	0.093	49.42	53.28	56.53	60.40	62.81	64.61	67.55	−1.546	56.143	0.096	48.76	52.79	56.14	60.10	62.53	64.33	67.26
12	−1.805	58.035	0.096	50.51	54.59	58.04	62.17	64.75	66.69	69.87	−1.547	58.019	0.099	50.16	54.44	58.02	62.27	64.90	66.85	70.03
13	−1.794	59.655	0.098	51.76	56.03	59.66	64.02	66.76	68.82	72.21	−1.548	59.889	0.101	51.64	56.12	59.89	64.38	67.16	69.23	72.62
14	−1.784	61.403	0.100	53.18	57.62	61.40	65.96	68.83	70.98	74.55	−1.549	61.525	0.102	53.00	57.63	61.53	66.17	69.05	71.20	74.71
15	−1.773	63.174	0.100	54.66	59.26	63.17	67.90	70.87	73.10	76.80	−1.550	62.727	0.101	54.08	58.78	62.73	67.43	70.35	72.52	76.08
16	−1.762	64.856	0.101	56.08	60.83	64.86	69.72	72.78	75.08	78.89	−1.551	63.554	0.100	54.86	59.59	63.55	68.27	71.20	73.37	76.92
17	−1.751	66.406	0.101	57.42	62.28	66.41	71.39	74.52	76.87	80.76	−1.552	64.146	0.100	55.43	60.17	64.15	68.87	71.79	73.96	77.51
18	−1.740	67.826	0.101	58.65	63.61	67.83	72.90	76.09	78.48	82.44	−1.553	64.545	0.099	55.81	60.57	64.55	69.27	72.19	74.36	77.90
19	−1.730	69.159	0.100	59.81	64.87	69.16	74.32	77.56	79.99	84.00	−1.554	64.837	0.099	56.09	60.85	64.84	69.56	72.49	74.65	78.19
WAIST to HEIGHT RATIO (WHtR) (*N* = 68,261)																				
5	−0.269	0.465	0.075	0.41	0.44	0.47	0.49	0.50	0.51	0.53	−0.724	0.460	0.079	0.41	0.44	0.46	0.49	0.50	0.51	0.53
6	−0.462	0.454	0.077	0.41	0.43	0.46	0.48	0.49	0.50	0.52	−0.793	0.449	0.080	0.40	0.43	0.45	0.47	0.49	0.50	0.52
7	−0.691	0.444	0.078	0.40	0.42	0.45	0.47	0.48	0.49	0.51	−0.863	0.439	0.082	0.39	0.42	0.44	0.46	0.48	0.49	0.51
8	−1.001	0.434	0.079	0.39	0.42	0.44	0.46	0.47	0.48	0.50	−0.932	0.430	0.084	0.38	0.41	0.43	0.46	0.47	0.48	0.50
9	−1.361	0.426	0.081	0.38	0.41	0.43	0.45	0.47	0.48	0.49	−1.002	0.422	0.086	0.37	0.40	0.42	0.45	0.46	0.47	0.49
10	−1.682	0.420	0.082	0.38	0.40	0.42	0.45	0.46	0.47	0.49	−1.071	0.417	0.089	0.36	0.39	0.42	0.44	0.46	0.47	0.49
11	−1.900	0.414	0.084	0.37	0.40	0.42	0.44	0.46	0.47	0.49	−1.141	0.413	0.092	0.36	0.39	0.41	0.44	0.46	0.47	0.49
12	−2.015	0.410	0.085	0.37	0.39	0.41	0.44	0.45	0.47	0.48	−1.210	0.412	0.094	0.36	0.39	0.41	0.44	0.46	0.47	0.49
13	−2.056	0.406	0.087	0.36	0.39	0.41	0.44	0.45	0.46	0.48	−1.279	0.412	0.097	0.36	0.39	0.41	0.44	0.46	0.47	0.49
14	−2.038	0.404	0.089	0.36	0.39	0.41	0.43	0.45	0.46	0.48	−1.349	0.413	0.099	0.36	0.39	0.41	0.44	0.46	0.47	0.50
15	−1.957	0.403	0.091	0.36	0.38	0.41	0.43	0.45	0.46	0.48	−1.418	0.415	0.100	0.36	0.39	0.41	0.45	0.46	0.48	0.50
16	−1.816	0.404	0.093	0.36	0.38	0.41	0.43	0.45	0.46	0.48	−1.487	0.417	0.100	0.36	0.39	0.42	0.45	0.47	0.48	0.50
17	−1.637	0.407	0.095	0.36	0.39	0.41	0.44	0.45	0.47	0.49	−1.556	0.420	0.100	0.36	0.39	0.42	0.45	0.47	0.48	0.51
18	−1.430	0.410	0.096	0.36	0.39	0.41	0.44	0.46	0.47	0.49	−1.626	0.423	0.100	0.37	0.40	0.42	0.45	0.47	0.49	0.51
19	−1.217	0.413	0.098	0.36	0.39	0.42	0.44	0.46	0.47	0.49	−1.695	0.425	0.100	0.37	0.40	0.43	0.46	0.48	0.49	0.52
BODY MASS INDEX (BMI) (*N* = 67,741)																				
5	−2.745	13.821	0.083	12.30	13.12	13.82	14.69	15.25	15.68	16.42	−2.128	13.595	0.087	12.01	12.87	13.59	14.47	15.02	15.43	16.11
6	−2.660	13.765	0.088	12.18	13.03	13.76	14.68	15.28	15.73	16.52	−2.063	13.578	0.092	11.90	12.81	13.58	14.51	15.10	15.54	16.28
7	−2.576	13.789	0.092	12.13	13.01	13.79	14.76	15.39	15.88	16.73	−1.998	13.631	0.098	11.85	12.81	13.63	14.63	15.27	15.76	16.56
8	−2.492	13.930	0.098	12.17	13.11	13.93	14.97	15.65	16.18	17.10	−1.933	13.797	0.105	11.89	12.91	13.80	14.89	15.59	16.12	17.02
9	−2.407	14.157	0.103	12.28	13.27	14.16	15.28	16.02	16.60	17.61	−1.869	14.071	0.113	12.00	13.11	14.07	15.27	16.05	16.65	17.66
10	−2.323	14.447	0.109	12.43	13.50	14.45	15.66	16.48	17.11	18.23	−1.804	14.457	0.121	12.20	13.40	14.46	15.79	16.66	17.33	18.48
11	−2.238	14.794	0.115	12.63	13.77	14.79	16.11	17.00	17.70	18.93	−1.739	14.985	0.128	12.52	13.82	14.98	16.46	17.43	18.19	19.50
12	−2.153	15.209	0.120	12.90	14.11	15.21	16.63	17.59	18.35	19.69	−1.674	15.664	0.134	12.98	14.40	15.66	17.28	18.36	19.19	20.64
13	−2.069	15.713	0.124	13.25	14.54	15.71	17.23	18.26	19.07	20.50	−1.608	16.444	0.138	13.55	15.08	16.44	18.19	19.34	20.24	21.80
14	−1.984	16.310	0.127	13.69	15.07	16.31	17.92	19.00	19.85	21.36	−1.543	17.208	0.139	14.15	15.77	17.21	19.04	20.25	21.19	22.81
15	−1.899	16.955	0.129	14.19	15.65	16.96	18.64	19.77	20.66	22.22	−1.478	17.823	0.138	14.65	16.33	17.82	19.70	20.93	21.88	23.50
16	−1.815	17.585	0.130	14.69	16.22	17.59	19.34	20.51	21.42	23.02	−1.413	18.271	0.136	15.03	16.76	18.27	20.16	21.39	22.34	23.94
17	−1.730	18.169	0.130	15.15	16.75	18.17	19.98	21.17	22.10	23.71	−1.348	18.607	0.135	15.32	17.08	18.61	20.50	21.72	22.65	24.22
18	−1.645	18.708	0.129	15.59	17.25	18.71	20.55	21.76	22.69	24.29	−1.283	18.884	0.133	15.57	17.34	18.88	20.78	21.99	22.90	24.43
19	−1.560	19.231	0.128	16.03	17.73	19.23	21.10	22.32	23.25	24.83	−1.218	19.140	0.132	15.79	17.59	19.14	21.03	22.23	23.13	24.62

Abbreviations: BMI, body mass index; WC, waist circumference; WHtR, waist‐to‐height‐ratio.

WHtR followed a different pattern with values declining initially and then increasing gradually at older ages. In boys, the WHtR decreased steadily till age 16 and then increased from age 17 years in percentiles ≥50th. In girls, the WHtR declined till age 13–15, thereafter the increase in WHtR set in earlier at higher centiles (age 14 at the 95th percentile, age 16 at the 85th percentile).

BMI values at the level of percentiles 5, 25, 50, 75. 85, 90, and 95, and LMS parameters are presented in appendix Table 1 and corresponding centile curves presented in Figure 2. BMI increased with age in both boys and girls; however, there were marked sex differences in the shape of the curves. Girls had lower BMI values than boys at younger ages (5–10 years), and higher BMI values thereafter. Among girls, there was a sharper increase in BMI between 11 and 14 years, then gradual plateauing from age 15 years. Boys exhibited a steady increase with a marginally higher increase between 11 and 15 years. There were no significant differences in centile curves and corresponding BMI values with the “reference” population sample 2 (Table S1, Figure S1). For sample 3, there were no differences at higher percentiles, but significant differences were observed at percentiles below the median (Table S3, Figure 2).

**FIGURE 3:**
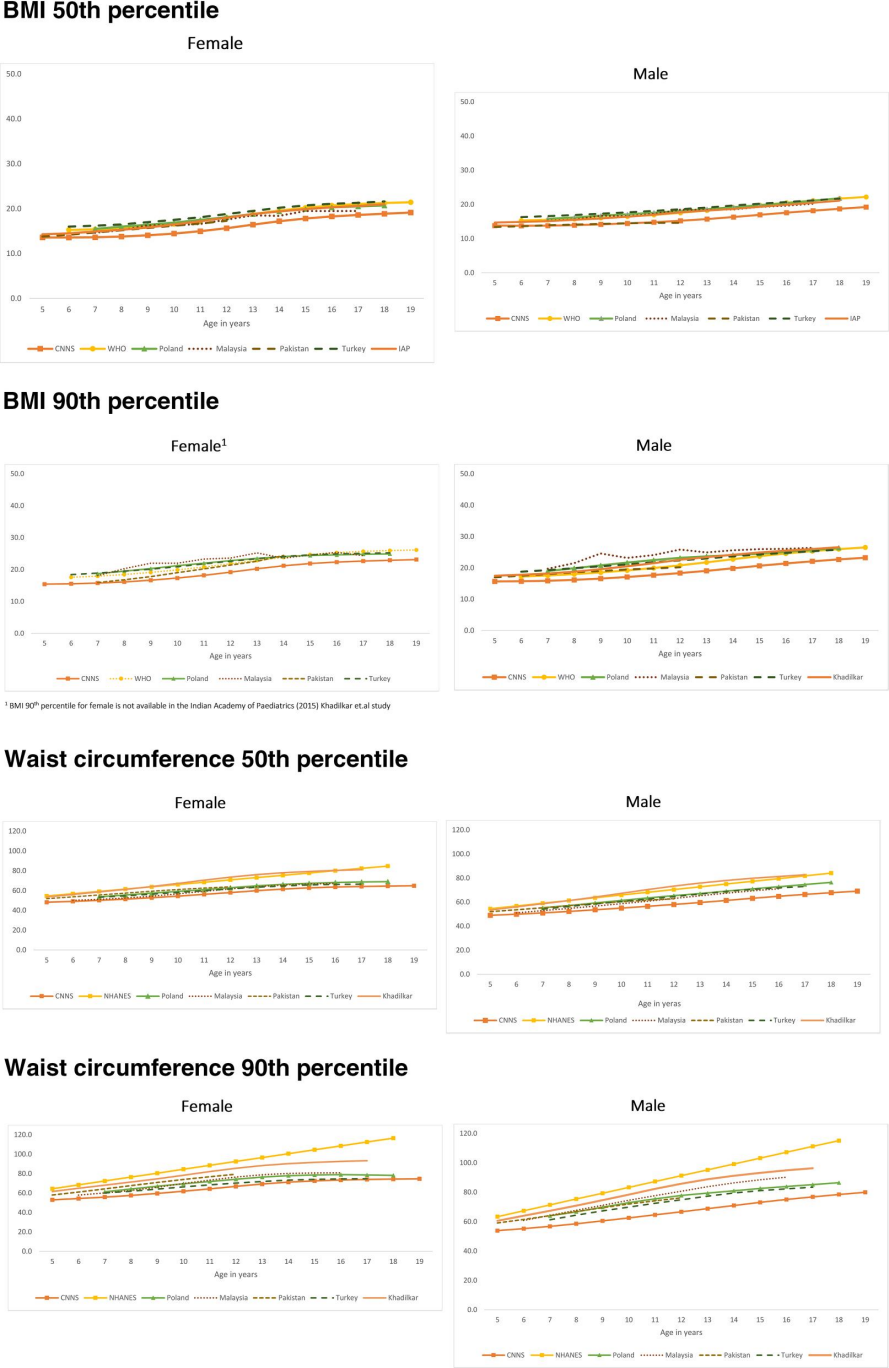
Comparison of the 50th and 90th percentile of waist circumference and BMI of children and adolescents from the CNNS (2016–2018), India (Khadlikar, 2015; Indian Academy of Pediatrics [IAP], 2015), US (WC: 2009–2014), Poland (WC: 2002–2005; BMI: 2007–2009), Malaysia (WC: 2008–2009; BMI: 2011), Pakistan (2009–2010), Turkey (2005), and WHO 2007 reference population BMI 90th percentile for female is not available in the Indian Academy of Paediatrics (2015) Khadilkar et.al study

The percentiles for WC from CNNS were lower than those from the US, Poland, Turkey, Malaysia, and Pakistan (Figure 3; Table S2). The WC centiles were also lower than those previously reported by from India by Khadlikar (2014). BMI centiles from CNNS were lower than those from Poland, Turkey, Malaysia, Pakistan (available for 5–12 years) and the WHO reference population (Figure 3, Table S3). Additionally, BMI percentiles were also lower than reported previously by the Indian Academy of Pediatrics (IAP, 2015).

### 3.2 Prevalence of obesity

The prevalence of BMI based general obesity using the WHO reference was 1.1% (95% CI: 1.0–1.3), marginally higher among males (Table 3). The prevalence of general obesity was significantly higher when based on the Indian population specific centiles (2.9%; 95% CI: 2.6–3.2); there was no difference between males and females. Central obesity based on WC ≥ 90th percentile was 6.1% (Male: 6.0%; 95% CI: 5.5–6.6 vs. Female: 6·.2%; 95% CI: 5.7–6.6; *p* = 0.095). 76% of the children identified under the general obesity (using India specific BMI centiles) were also identified as having central obesity (Table 2). Central obesity based on WHtR ≥ 0.5 was 5.3% (95% CI: 5.0–5.7); the prevalence was higher among females (Female: 5.7%; 95% CI: 5.2–6.3 vs. Male: 4.9%, 95% CI: 4.5–5.4; *p* = 0.067). 69% of the children identified under general obesity (India specific BMI centiles) were also identified as centrally obese based on WHtR. The WHtR cutoff of 0.5 corresponded to the 92nd percentile for males and for females. The combined classification of central obesity (WC ≥ 90th centile and WHtR ≥ 0·5) yielded a prevalence of 3.6% (95% CI: 3.3–3.9), higher among girls (Female: 3.9%; 95% CI: 3.5–4.4 vs. Male: 3.2%; 95% CI: 2.9–3.6; *p* = 0·.11). 65% of children identified as having general obesity (India specific centiles) were also identified as centrally obese by this criterion.

**TABLE 3:** Prevalence of general and central obesity among male and female children 5–19 years of age, India, 2016–2018

Variable	Male (*N* = 35,449) %	Female (*N* = 32,812) %	Total (*N* = 68,261) %	Overlap with children/adolescents with general obesity (India Specific Centiles) %
General obesity based on BMI^a^				
BMI =>2SD^b^ (WHO reference population)	1.3 (1.1–1.5)	1 (0.8–1.2)	1.1 (1.0–1.3)	40.0 (36.5–43.1)
BMI =>2SD^c^ (Indian population specific centiles)	2.9 (2.6–3.3)	2.8 (2.5–3.2)	2.9 (2.6–3.2)	‐
Central obesity based on WC^d^				
WC ≥ 90th percentile	6.0 (5.5–26.6)	6.2 (5.7–6.8)	6.1 (5.7–6.6)	76.3 (72.9–79.4)
Central obesity based on WHtR^e^				
WtHR ≥ 0.5	4.9 (4.5–5.4)	5.7 (5.2–6.3)	5.3 (5.0–5.7)	69.2 (65.8–72.4)
Central obesity based on WC & WHtR^d^ ^,^ ^e^				
Central obesity (WC ≥ 90 percentile and WtHR ≥ 0.5)	3.2 (2.9–3.6)	3.9 (3.5–4.4)	3.6 (3.3–3.9)	65.4 (61.9–68.8)

Abbreviations: BMI, body mass index; WC, waist circumference; WHtR, waist‐to‐height‐ratio.

^a^ 520 cases with BMI not measured were excluded. Male *N* = 35,130; Female *N* = 32,611; Total *N* = 67,741.

^b^ Age‐ and sex‐specific BMI =>2SD *z*‐score cutoff values generated from WHO reference applied to newly constructed CNNS percentiles for sample 1 (all eligible participants).

^c^ Age‐ and sex‐specific BMI =>2SD *z*‐score values from newly constructed CNNS percentiles for sample 1 (all eligible participants).

^d^ WC ≥ 90th percentile from newly constructed CNNS percentiles for sample 1 (all eligible participants).

^e^ Fixed waist‐to‐height ratio from sample 1 (all eligible participants).

Table 4 shows the mean and standard deviation of WC, WHtR, and BMI *z*‐scores across socio‐demographic characteristics of the study population. For all three measures—WC, WHtR, and BMI—the mean *z*‐scores were significantly higher (*p* < 0.001) in urban subjects, in those who were economically better off; those from higher castes; those with educated mothers and those with access to safe sanitation.

**TABLE 4:** WC, WtHR, and BMI *z* scores by background characteristics of children and adolescents aged 5–19 years in India (2016–2018)

Background characteristics	*n*	Waist circumference (WC) *z*‐score		Waist to height ratio (WHtR) *z*‐score		*n*	BMI z‐score	
		Mean (SD)	*p*‐value*	Mean (SD)	*p*‐value*		Mean (SD)	*p*‐value*
Sex			<0.001		0.7702			0.001
Male	35,449	0.13 (1.12)		0.09 (1.12)		35,125	0.27 (1.08)	
Female	32,812	0.15 (1.12)		0.09 (1.11)		32,607	0.24 (1.09)	
Place of residence			<0.001		<0.001			<0.001
Rural	35,347	0.04 (1.05)		0.10 (1.01)		37,325	0.14 (1.02)	
Urban	28,915	0.26 (1.18)		0.19 (1.12)		30,407	0.39 (1.13)	
Wealth index			<0.001		<0.001			<0.001
Poorest	5167	−0.08 (0.93)		0.10 (0.94)		5500	−0.09 (0.92)	
Poor	7920	−0.05 (1.01)		0.06 (1.03)		8409	0.02 (1.00)	
Middle	11,937	−0.02 (1.05)		0.05 (1.05)		12,633	0.13 (1.02)	
Rich	16,668	0.10 (1.10)		0.06 (1.09)		17,545	0.24 (1.06)	
Richest	22,570	0.36 (1.20)		0.13 (1.20)		23,645	0.49 (1.15)	
Caste			<0.001		<0.001			<0.001
SC/ST	24,813	0.11 (1.07)		0.14 (1.07)		26,019	0.27 (1.05)	
OBC	20,164	0.06 (1.12)		−0.02 (1.10)		21,454	0.14 (1.08)	
Others	19,285	0.26 (1.16)		0.13 (1.14)		20,259	0.35 (1.12)	
Religion			<0.001		<0.001			<0.001
Hindu	44,239	0.09 (1.12)		0.03 (1.10)		46,884	0.18 (1.09)	
Muslim	8290	0.13 (1.12)		0.04 (1.11)		8788	0.17 (1.08)	
Christian	8189	0.32 (1.05)		0.37 (1.06)		8404	0.64 (0.98)	
Sikh	1277	0.17 (1.17)		−0.09 (1.16)		1322	0.44 (1.05)	
Others	2267	0.37 (1.05)		0.39 (1.02)		2334	0.57 (0.99)	
Mothers' education			<0.001		<0.001			<0.001
No school	19,944	0.02 (1.02)		0.12 (0.99)		21,063	0.07 (0.99)	
Primary	7669	0.01 (1.06)		0.13 (1.00)		8111	0.15 (1.03)	
Secondary	21,776	0.11 (1.13)		0.11 (1.07)		22,936	0.27 (1.09)	
Higher	14,873	0.39 (1.21)		0.21 (1.16)		15,622	0.52 (1.16)	
Safe sanitation			<0.001		<0.001			<0.001
No	25,252	0.01 (1.04)		0.03 (1.04)		26,847	0.07 (1.03)	
Yes	39,010	0.22 (1.16)		0.12 (1.14)		40,885	0.37 (1.10)	
Safe drinking water			0.013		0.028			0.031
No	8336	0.15 (1.14)		0.11 (1.12)		8797	0.28 (1.09)	
Yes	55,835	0.13 (1.12)		0.08 (1.10)		58,841	0.25 (1.08)	

Abbreviations: BMI, body mass index; WC, waist circumference; WHtR, waist‐to‐height‐ratio.

*ANOVA is used for statistical significance.

## 4 DISCUSSION

This paper presents age‐ and sex‐specific WC, WHtR, and BMI percentile curves drawn from a nationally representative population of children and adolescents (5–19 years) in India with strong emphasis on quality control and monitoring. In conformity with earlier Indian and global studies, girls had lower BMI values than boys at younger ages, and higher BMI values beyond 11–12 years of age.^19^ ^,^ ^28^, ^29^, ^30^ The study found substantially lower BMI values for boys and girls compared to the IAP (2015)^19^ and the WHO 2007 reference population.^5^ Girls had lower WC values than boys at all ages. The pattern of the percentile curves is similar to that previously reported from India by Khadlikar et al. (2014) but with substantially lower percentile values.^18^ WC centile values are also lower than those reported by Kuriyan et al. (2011) from South India.^33^ Data for both the IAP (BMI) and Khadlikar (WC) references are drawn from urban, school‐going children from middle‐to‐higher economic strata who may already be tending towards overweight. In contrast, the CNNS percentiles are drawn from a nationally representative population including both rural and urban settings, and reveal a population that is less obese. The construction of normative data from population surveys, and whether such normative data can be used to assess prevalence of extreme measurements (obesity in this case) have been the subject of debate—it was suggested that normative data should be drawn from children who are in the best of health and have no constraints related to growth adversaries such as nutrition and socio‐economic factors. Nationally representative surveys have been used previously for constructing percentiles and defining obesity in children.^34^, ^35^ WHO has highlighted concerns about using descriptive samples of populations that reflect a secular trend towards overweight and obesity to construct growth references may result in an upward skewness of data with overestimation of undernutrition and underestimation of overweight and obesity.^36^, ^37^ Sensitivity analysis comparing the full sample of eligible participants and that excluding thin and obese (±2SD) participants showed no difference in 90th percentiles values for WC and 95th percentile values for BMI used to define general and central obesity; at the same time, however, there were differences at the lower percentiles with higher percentile values that would substantially overestimate underweight—this is similar to the concerns expressed above. In comparison with several international studies, the CNNS percentiles centiles were substantially lower than those from the US (WC),^27^ Malaysia (WC and BMI),^10^, ^28^ Poland (WC and BMI),^11^, ^29^ Turkey (WC and BMI),^17^, ^30^ Pakistan (WC and BMI available only for 5–12 years),^9^, ^38^ and the WHO reference population (BMI).^5^ These findings must be interpreted with caution as data collection occurred at different time points in these countries—several years prior to the CNNS—and not from nationally representative samples. Furthermore, genetic and environmental factors differ across geographies and ethnicities. These geographic variations highlight the relevance of country specific centile curves and the need for surveys with a nationally representative population to document any secular trends in these anthropometric metrics. We are constrained by the fact that we do not have any appropriate comparison group within the country or internationally with nationally representative data collected in recent years.

The prevalence of BMI‐based general obesity in CNNS data (2.9%) was higher than that derived from BMI *z*‐scores using the WHO reference population (1.1%)—this is along expected lines, as the global reference includes data from western nations including the US, and is likely to underestimate obesity in children from developing countries.^15^ The prevalence is close to that reported by the Global Burden of Disease study^3^ that examined prevalence estimates between 1981 and 2013, but is significantly lower than estimates reported by Ranjani et al.^4^ from 42 smaller studies between 2010 and 2013 (only urban prevalence estimates were included in trend analyses). The prevalence of central obesity based on the combined WC ≥ 90th percentile and WHtR ≥ 0.5 indicator, was higher than general obesity. Used singly, WC ≥ 90th and WHtR yielded significantly higher prevalence of central obesity. This finding highlights the divergence between general and central obesity in Indian children, and the higher prevalence of central obesity. The prevalence using the WC ≥ 90thC cutoff closely mirrored the prevalence derived from the WHtR cutoff. WHtR, being a ratio of WC and height, removes distortions based on body frame size in different populations, and studies have shown it to be superior to BMI or percentage body fat in predicting cardio‐metabolic risk.^14^, ^31^ There was a significant overlap between general and central obesity: 76% of children identified as having general obesity were also identified to have central obesity based on the WC ≥ 90th percentile, 69% children based on WHtR ≥ 0.5 and 65% based on the combined criteria of WC ≥ 90thC and WHtR ≥ 0.5. The choice of cutoff value to identify obesity is critical. The cutoff of WC ≥ 90th percentile identifies both central obesity and three‐quarters of general obesity, and could, therefore, serve as a single useful measure; similarly for WHtR ≥ 0.5. The more conservative combined WC and WHtR indicator provides a lower estimate of obesity that can also identify two‐thirds of the children with general obesity. The widest net for identifying obese children would be cast by using both BMI and WC, or WHtR criteria. The 2020 Consensus Statement on WC recommends the use of WC in addition to BMI to assess obesity.^8^ It has been observed that both BMI and WC,or WHtR perform similarly when predicting a cluster of cardio‐metabolic risk factors, with greater effect seen among obese children.^39^ Further analyses are needed to assess the relative utility of BMI, WC, and WHtR cut‐offs used in this survey for predicting associated cardio‐metabolic risk factors.

The study found lower age‐ and sex‐specific *z*‐score values for BMI, WC, and WHtR in rural areas and in poorer households, suggesting that at a national level obesity exists largely in well‐off urban pockets. Other indicators pointing towards an association between wealth and overweight/obesity were higher *z*‐scores for BMI, WC, and WHtR in households with better sanitation, higher educational attainment of mothers, and higher caste. This is further supported by evidence from the comparison of centile values from economically better off study populations and the CNNS data, reported above. Changes in lifestyle with urbanization including reduced physical activity, increased sedentary living, and unhealthy diets may be probable underlying causes. Mushtaq and colleagues reported similar findings from Pakistan, a country with an ethnically similar population to India.^9^

In conclusion, the conventional metrics recommended for identifying children with general or central obesity are consistently lower with the nationally representative CNNS reference than several international references including that from the WHO. This suggests that Indian children and adolescents are relatively thinner which could be due to a combination of genetic, environmental, and inter‐generational factors. It is therefore possible that prediction of cardio‐metabolic risk factors associated with central or general obesity would be lower if international cut‐offs were employed. Further analyses are required to determine cutoffs associated with biomarker‐based cardio‐metabolic risk factors in this population. Finally, the nationally representative reference will prove invaluable for documenting and comparing the details, especially the distribution of secular trends in this population.

## AUTHOR CONTRIBUTIONS

Avina Sarna, Akash Porwal, Sowmya Ramesh, and Harshpal Singh Sachdev conceptualized the manuscript. Avina Sarna and Harshpal Singh Sachdev designed the survey, Nizamuddin Khan did data quality control and computed sampling weights. Akash Porwal led the statistical analyses and Sana Ashraf contributed to analyses. Avina Sarna, Harshpal Singh Sachdev, Rajib Acharya, and Sikha Sinha guided the analysis and interpreted the results. Avina Sarna led the writing of the manuscript with inputs from Akash Porwal, Rajib Acharya, and Sowmya Ramesh. Harshpal Singh Sachdev reviewed the manuscript.

## Supporting information

Supplementary Material

## References

[1] Expert panel on integrated guidelines for cardiovascular health and risk reduction in children and adolescents: national heart, lung and blood Institute. Expert panel on integrated guidelines for cardiovascular health and risk reduction in children and adolescents: summary report. Pediatrics. 2011;128(Suppl 5):S213–S256. 22084329 10.1542/peds.2009-2107C PMC4536582

[2] Singh A , Mulder C , Twisk J , vanMechelen W , Chinapaw M . Tracking of childhood overweight into adulthood: a systematic review of the literature. Obes Rev. 2008;9:474–488. 18331423 10.1111/j.1467-789X.2008.00475.x

[3] Ng M , Fleming T , Robinson M , et al. Global, regional, and national prevalence of overweight and obesity in children and adults during 1980–2013: a systematic analysis for the Global Burden of Disease Study 2013. Lancet. 2014;384(9945):766–781. 24880830 10.1016/S0140-6736(14)60460-8 PMC4624264

[4] Ranjani H , Mehreen TS , Pradeepa R , et al. Epidemiology of childhood overweight & obesity in India: a systematic review. Indian J Med Res. 2016;143(2):160–174. 27121514 10.4103/0971-5916.180203 PMC4859125

[5] WHO . World Health Organization growth reference data for 5‐19 years. 2007. https://www.who.int/groWHtRef/en/. Accessed February 6, 2020.

[6] WHO . Waist Circumference and Waist‐Hip Ratio: Report of a WHO Expert Consultation, 8–11 December 2008. Geneva: World Health Organization; 2011.

[7] Deepa M , Farooq S , Deepa R , Manjula D , Mohan V . Prevalence and significance of generalized and central body obesity in an urban Asian Indian population in Chennai, India (CURES: 47). Eur J Clin Nutr. 2007;63:259–267. 17928807 10.1038/sj.ejcn.1602920

[8] Ross R , Neeland I , Yamashita S , et al. Waist circumference as a vital sign in clinical practice: a Consensus statement from the IAS and ICCR working group on visceral obesity. Nat Rev Endocrinol. 2020;16(3):177–189. 32020062 10.1038/s41574-019-0310-7 PMC7027970

[9] Mushtaq M , Gull S , Abdullah H , Shahid U , Shad M , Akram J . Waist circumference, waist‐hip ratio and waist height ratio percentiles and central obesity among Pakistani children aged five to twelve years. BMC Pediatr. 2011;11:105. 22104025 10.1186/1471-2431-11-105 PMC3239239

[10] Poh B , Jannah A , Chong L , Ruztia A , Ismail M , McCarthy D . Waist circumference percentile curves for Malaysian children and adolescents aged 6.0–16.9 years. Int J Pediatr Obes. 2011;6:229–235. 21668385 10.3109/17477166.2011.583658

[11] Nawarycz O , Krzyzaniak A , Stawińska‐Witoszyńska B , et al. Percentile distributions of waist circumference for 7–19 year‐old Polish children and adolescents. Obes Rev. 2010;11(4):281–288. 20003070 10.1111/j.1467-789X.2009.00694.x

[12] Ashwell M . Waist to height ratio and the AshwellR shape chart could predict the health risks of obesity in adults and children in all ethnic groups. Nutr Food Sci. 2005;35:359–364.

[13] McCarthy H , Ashwell M . A study of central fatness using waist‐to‐height ratios in UK children and adolescents over two decades supports the simple message—'keep your waist circumference to less than half your height'. Int J Obes. 2006;30:988–992. 10.1038/sj.ijo.0803226 16432546

[14] Patel S , Deepa M , Shivashankar R , et al. Comparison of multiple obesity indices for cardiovascular disease risk classification in South Asian adults: the CARRS Study. 2017;12(4):e0174251. 10.1371/journal.pone.0174251 PMC5407781 28448582

[15] Deurenberg P , Deurenberg‐Yap M , Guricci S . Asians are different from Caucasians and from each other in their body mass index/body fat per cent relationship. Obes Rev. 2002;3:141–146. 12164465 10.1046/j.1467-789x.2002.00065.x

[16] Misra A , Shah P , Goel K , et al. The high burden of obesity and abdominal obesity in urban Indian schoolchildren: a multicentric study of 38,296 children. Ann Nutr Metab. 2011;58(3):203–211. 21757894 10.1159/000329431

[17] Hatipoglu N , Ozturk A , Mazicioglu M , Kurtoglu S , Seyhan S , Lokoglu F . Waist circumference percentiles for 7‐ to 17‐year‐old Turkish children and adolescents. Eur J Pediatr. 2008;167:383‐389. 17487506 10.1007/s00431-007-0502-3

[18] Khadlikar A , Ekbote V , Chiplonkar S , et al. Waist circumference percentiles in 2–18 year old Indian children. J Pediatr. 2014;164(6):1358–1362. 24655536 10.1016/j.jpeds.2014.02.018

[19] Khadlikar V , Yadav S , Agarwal K , et al. Revised IAP growth charts for height, weight and body mass index for 5‐ to 18‐year‐old Indian children. Indian Pediatr. 2015;52:47–55. 25638185 10.1007/s13312-015-0566-5

[20] Khadilkar V , Khadilkar A , Cole T , Chiplonkar S , Pandit D . Overweight and obesity prevalence and body mass index trends in Indian children. Int J Pediatr Obes. 2011;6(2–2):e216‐e224. 21158695 10.3109/17477166.2010.541463

[21] Ministry of Health and Family Welfare (MoHFW) , Government of India , UNICEF and Population Council . Comprehensive National Nutrition Survey (CNNS) National Report. New Delhi; 2019. https://nhm.gov.in/showfile.php?lid=712

[22] Centers for Disease Control and Prevention (CDC) . National Center for Health Statistics (NCHS). Anthropometry Procedures Manual. Hyattsville, MD; U.S. Department of Health and Human Services, Centers for Disease Control and Prevention. 2004. https://www.cdc.gov/nchc/data/nhanes/nhanes_03_04/BM.pdf. Accessed March 2015.

[23] Ulijaszek S , Kerr D . Anthropometric measurement error and the assessment of nutritional status. Br J Nutr. 1999;82(3):165–177.Erratum in: Br J Nutr. 2000 Jan; 83(1)95. PMID 10655963. 10.1017/s0007114599001348. 10655963

[24] Cole T , Green P . Smoothing reference centile curves: the LMS method and penalized likelihood. Stat Med. 1992;11(10):1305‐1319. 1518992 10.1002/sim.4780111005

[25] Stasinopoulos D , Robert A . Generalized Additive models for location scale and shape (GAMLSS) in R. Journal of Statistical software. J Stat Softw. 2007;23(7).

[26] Royston P , Weight E . Goodness‐of‐fit statistics for age‐specific reference intervals. Stat Med. 2000;19:2943‐2962. 11042625 10.1002/1097-0258(20001115)19:21<2943::aid-sim559>3.0.co;2-5

[27] Fernández J , Brown M , López‐Alarcón M , et al. Changes in pediatric waist circumference percentiles despite reported pediatric weight stabilization in the United States. Pediatr Obes. 2017;12(5):347–355. 27273320 10.1111/ijpo.12150 PMC5145787

[28] Bonn, B , Shariff, A , Mohamed, A , Merican, A . Proceeding of the International Conference on Mathematics and Sciences (ICOMSc) 2011. ISB5 N 978‐602‐19142‐0‐5.

[29] Kułaga Z , Litwin M , Tkaczyk M , et al. Polish 2010 growth references for school‐aged children and adolescents. Eur J Pediatr. 2011;170:599–609. 20972688 10.1007/s00431-010-1329-x PMC3078309

[30] Ozturk A , Mazicioglu M , Hatipoglu N , et al. Reference body mass index curves for Turkish children 6 to 18 years of age. J Pediat Endocrinol & Metab. 2008;21:827–836. 10.1515/jpem.2008.21.9.827 18924576

[31] Fernandez J , Redden D , Pietrobelli A , Allison D . Waist circumference percentiles in nationally representative samples of African‐American, European‐American, and Mexican‐American children and adolescents. J Paediatr. 2004;145:439–44. 10.1016/j.jpeds.2004.06.044 15480363

[32] Nambiar S , Hughes I , Davies P . Developing waist‐to‐height ratio cut‐offs to define overweight and obesity in children and adolescents. Publ Health Nutr. 2010;13(10):1566–1574. 10.1017/S1368980009993053 20100388

[33] Kuriyan R , Thomas T , Lokesh D , et al. Waist circumference and waist for height percentiles in urban South Indian children aged 3–16 years. Indian Pediatr. 2011;48:765–771. 21555800 10.1007/s13312-011-0126-6

[34] Cole T , Bellizzi M , Flegal K , Dietz W . Establishing a standard definition for child overweight and obesity worldwide: international survey. BMJ. 2000;320:1–6. 10797032 10.1136/bmj.320.7244.1240 PMC27365

[35] Song P Li X , Gasevic D , Flores A , Yu Z . BMI, waist circumference reference values for Chinese school‐aged children and adolescents. Int J Environ Res Public Health. 2016;13:589. 10.3390/ijerph13060589 PMC4924046 27314368

[36] DeOnis M , Onyango A , Borghi E , Siyam A , Nishida C , Siekmann J . Development of a WHO growth reference for school‐aged children and adolescents. Bull World Health Organ. 2007;85(9):649–732. www.who.int/bulletin/volumes/85/9/07‐043497/en/. 10.2471/BLT.07.043497 PMC2636412 18026621

[37] DeOnis M . The use of anthropometry in the prevention of childhood overweight and obesity. Int J Obes Relat Metab Disord. 2004;28:S81–S85. 15543225 10.1038/sj.ijo.0802810

[38] Mushtaq M , Gull S , Mushtaq K , et al. Height, weight and BMI percentiles and nutritional status relative to the international growth references among Pakistani school‐aged children. BMC Pediatr. 2012;12(31). 10.1186/1471-2431-12-31 PMC3337223 22429910

[39] Sardinha L , Santos D , Silva A , Grøntved A , Andersen L , Ekelund U . A comparison between BMI, waist circumference, and waist‐to‐height ratio for identifying cardio‐metabolic risk in children and adolescents. PLoS One 11. 2016(2). e0149351. 10.1371/journalpone0149351. 26901828 PMC4762486

