# Supplementary material for: Waist circumference, waist‐to‐height ratio and BMI percentiles in children aged 5 to 19 years in India: A population‐based study
Source: Obes Sci Pract. 2021 Mar 23;7(4):392–404. doi: 10.1002/osp4.493 (PMC8346379; doi:10.1002/osp4.493)
Supplement: Supplementary file 1 — Supplementary Material [file OSP4-7-392-s001.pdf]

APPENDIX TABLES AND FIGURES

Appendix Table 1: Characteristics of a population-based sample of children and adolescents 5-19 years in India (2016-18)

Characteristics	weight (kg)		Height (cm)		Waist circumference (cm)		Waist circumference height ratio		Body mass index	
	(n)	Mean (SD)	(n)	Mean (SD)	(n)	Mean (SD)	(n)	Mean (SD)	(n)	Mean (SD)
**Boys**	**35390**	**33.0 (14.4)**	**35449**	**139.6 (20.9)**	**35449**	**59.0 (8.9)**	**35449**	**0.42 (0.04)**	**35130**	**16.0 (2.9)**
5	3624	16.4(6.3)	3640	106.6(6.2)	3640	49.8(3.6)	3640	0.47(0.04)	3582	14.0(1.4)
6	3839	17.8(3.7)	3848	112.5(6.7)	3848	50.6(3.8)	3848	0.45(0.04)	3796	14.0(1.5)
7	3869	19.9(4.9)	3872	118.5(6.8)	3872	52.1(4.4)	3872	0.44(0.03)	3831	14.1(1.5)
8	4117	21.9(4.5)	4126	123.5(7.4)	4126	53.2(4.8)	4126	0.43(0.04)	4081	14.3(1.8)
9	3585	24.5(6.6)	3589	128.6(7.6)	3589	54.9(5.4)	3589	0.43(0.04)	3556	14.6(2.0)
10	1837	27.1(7.3)	1838	133.5(7.4)	1838	56.4(5.6)	1838	0.42(0.04)	1821	15.0(2.1)
11	1766	29.8(6.9)	1769	138.6(8.3)	1769	58.1(6.7)	1769	0.42(0.04)	1754	15.4(2.5)
12	1867	32.7(7.7)	1868	143.3(9.6)	1868	59.3(6.3)	1868	0.41(0.04)	1854	15.8(2.3)
13	1819	37.0(8.9)	1821	149.5(9.4)	1821	61.6(7.3)	1821	0.41(0.04)	1816	16.4(2.6)
14	1860	41.3(9.0)	1860	155.4(9.6)	1860	63.0(6.8)	1860	0.41(0.04)	1851	17.0(2.5)
15	1865	45.6(9.6)	1868	159.7(8.8)	1868	64.8(7.2)	1868	0.41(0.04)	1860	17.7(2.7)
16	1765	48.7(9.4)	1766	162.9(8.0)	1766	66.6(8.1)	1766	0.41(0.05)	1759	18.3(2.9)
17	1558	51.5(9.0)	1562	164.4(7.4)	1562	68.1(7.5)	1562	0.41(0.04)	1555	19.0(2.8)
18	1801	52.7(9.2)	1803	165.3(7.2)	1803	69.2(7.3)	1803	0.42(0.04)	1797	19.2(2.7)
19	218	52.6(8.3)	219	164.1(7.2)	219	70.1(7.0)	219	0.43(0.04)	217	19.5(2.5)
**Girls**	**32766**	**32.1 (12.5)**	**32812**	**136.2 (17.5)**	**32812**	**58.0 (8.4)**	**32812**	**0.43 (0.04)**	**32611**	**16.3 (3.2)**
5	3476	15.5(2.9)	3486	105.7(6.8)	3486	48.8(3.6)	3486	0.46(0.04)	3455	13.8(1.4)
6	3635	17.2(3.4)	3642	111.4(6.6)	3642	49.9(3.8)	3642	0.45(0.04)	3610	13.8(1.5)
7	3556	19.2(3.9)	3565	117.5(6.7)	3565	50.9(4.0)	3565	0.43(0.03)	3525	13.8(1.6)
8	3642	21.5(5.2)	3646	122.5(7.3)	3646	52.4(5.5)	3646	0.43(0.04)	3619	14.2(1.8)
9	3046	23.8(4.9)	3049	127.8(7.6)	3049	53.9(5.5)	3049	0.42(0.04)	3032	14.5(2.0)
10	1612	26.8(5.9)	1613	133.3(8.4)	1613	55.8(5.7)	1613	0.42(0.04)	1604	15.0(2.2)
11	1686	30.2(7.0)	1686	138.7(8.1)	1686	57.3(6.4)	1686	0.41(0.04)	1683	15.6(2.5)
12	1777	34.0(8.2)	1777	143.4(8.0)	1777	59.5(6.3)	1777	0.42(0.04)	1769	16.3(2.6)
13	1714	37.8(8.7)	1718	147.1(7.6)	1718	61.5(7.0)	1718	0.42(0.04)	1708	17.4(2.9)
14	1661	41.7(9.3)	1663	150.7(6.5)	1663	63.9(7.6)	1663	0.42(0.05)	1656	18.3(3.1)
15	1874	42.1(6.7)	1876	151.7(6.1)	1876	63.4(6.3)	1876	0.42(0.04)	1868	18.3(2.6)
16	1751	43.7(6.9)	1752	152.5(6.0)	1752	64.3(6.3)	1752	0.42(0.04)	1749	18.8(2.6)
17	1553	44.8(8.2)	1556	152.7(6.0)	1556	65.7(7.4)	1556	0.43(0.05)	1551	19.2(3.1)
18	1568	44.8(7.1)	1568	152.3(5.8)	1568	65.4(6.8)	1568	0.43(0.04)	1567	19.3(2.7)
19	215	44.2(7.9)	215	151.2(5.8)	215	64.7(6.9)	215	0.43(0.04)	215	19.3(2.9)
**Total**	**68156**	**32.3 (13.5)**	**68261**	**137.9 (19.4)**	**68261**	**58.5 (8.7)**	**68261**	**0.43 (0.04)**	**67741**	**16.2 (3.1)**
5	7100	16.0(4.9)	7126	106.2(6.5)	7126	49.3(3.6)	7126	0.47(0.04)	7037	13.9(1.4)
6	7474	17.5(3.6)	7490	112.0(6.7)	7490	50.3(3.8)	7490	0.45(0.04)	7406	13.9(1.5)
7	7425	19.5(4.4)	7437	117.9(6.8)	7437	51.4(4.3)	7437	0.44(0.03)	7356	14.0(1.5)

For Review Only

8	7759	21.7(4.8)	7772	123.0(7.3)	7772	52.8(5.2)	7772	0.43(0.04)	7700	14.2(1.8)
9	6631	24.2(5.8)	6638	128.2(7.6)	6638	54.4(5.5)	6638	0.42(0.04)	6588	14.6(2.0)
10	3449	26.9(6.6)	3451	133.4(7.9)	3451	56.1(5.7)	3451	0.42(0.04)	3425	15.0(2.1)
11	3452	30.0(7.0)	3455	138.6(8.2)	3455	57.7(6.6)	3455	0.42(0.04)	3437	15.5(2.5)
12	3644	33.3(7.9)	3645	143.3(8.9)	3645	59.4(6.3)	3645	0.41(0.04)	3623	16.1(2.5)
13	3533	37.4(8.8)	3539	148.3(8.7)	3539	61.6(7.2)	3539	0.42(0.04)	3524	16.9(2.8)
14	3521	41.5(9.1)	3523	153.0(8.5)	3523	63.4(7.2)	3523	0.42(0.05)	3507	17.7(2.9)
15	3739	43.7(8.3)	3744	155.4(8.4)	3744	64.0(6.8)	3744	0.41(0.04)	3728	18.1(2.7)
16	3516	46.2(8.6)	3518	157.6(8.7)	3518	65.4(7.3)	3518	0.42(0.04)	3508	18.6(2.8)
17	3111	48.0(9.2)	3118	158.2(8.9)	3118	66.8(7.6)	3118	0.42(0.05)	3106	19.1(3.0)
18	3369	48.9(9.1)	3371	159.0(9.3)	3371	67.3(7.3)	3371	0.42(0.04)	3364	19.3(2.7)
19	433	48.4(9.1)	434	157.8(9.2)	434	67.5(7.4)	434	0.43(0.04)	432	19.4(2.7)

For Review Only

Appendix Figure 1: Smoothed waist circumference (WC) and BMI percentiles curves for males and females aged 5-19 years based on a "Reference" population in analytical sample 2 (removing ±3SD BMI z scores)

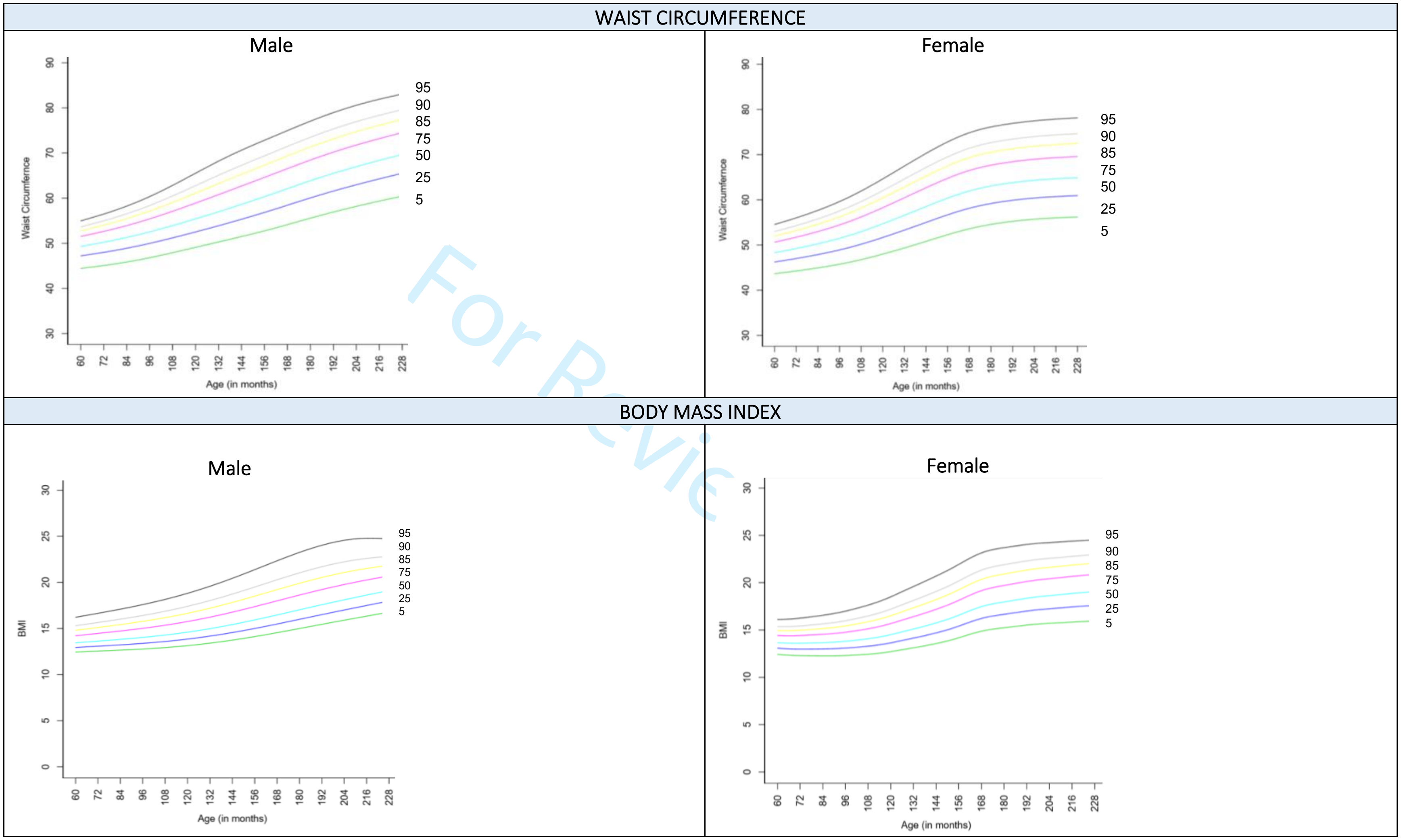

Excluded sample: 7.7% of boys and 4.1% of girls were thin (BMI < -3SD). Similarly, 0.1% of boys and 0.2% of girls were obese (BMI > -3SD).

For Revie

Appendix Table 2: Smoothed percentile values of WC (cm) and BMI by age and sex for males and females aged 5-19 years based on a “Reference” population in analytical sample 2 (removing ±3SD BMI z scores)

WAIST CIRCUMFERENCE (WC) (N=64,262)																				
Age (Years)	Males										Females									
	L	M	S	P5	P25	P50	P75	P85	P90	P95	L	M	S	P5	P25	P50	P75	P85	P90	P95
5	-0.445	49.306	0.065	44.45	47.22	49.31	51.52	52.77	53.64	54.97	-1.665	48.310	0.067	43.67	46.25	48.31	50.63	52.01	52.99	54.56
6	-0.707	50.237	0.068	45.08	48.01	50.24	52.65	54.02	54.99	56.48	-1.658	49.228	0.071	44.26	47.02	49.23	51.73	53.23	54.31	56.02
7	-1.040	51.294	0.072	45.85	48.91	51.29	53.93	55.45	56.54	58.23	-1.650	50.269	0.075	44.95	47.89	50.27	52.99	54.61	55.79	57.67
8	-1.475	52.530	0.076	46.82	49.99	52.53	55.42	57.14	58.38	60.35	-1.643	51.489	0.079	45.76	48.92	51.49	54.45	56.24	57.54	59.62
9	-1.942	53.923	0.080	47.93	51.21	53.92	57.11	59.06	60.50	62.84	-1.636	52.972	0.084	46.77	50.18	52.97	56.22	58.19	59.63	61.95
10	-2.290	55.419	0.085	49.11	52.52	55.42	58.91	61.12	62.78	65.53	-1.628	54.709	0.089	47.99	51.67	54.71	58.27	60.44	62.04	64.63
11	-2.415	56.989	0.089	50.30	53.90	56.99	60.79	63.22	65.08	68.20	-1.621	56.589	0.093	49.36	53.31	56.59	60.46	62.85	64.61	67.48
12	-2.316	58.632	0.092	51.50	55.33	58.63	62.69	65.30	67.28	70.63	-1.614	58.515	0.096	50.81	55.01	58.52	62.68	65.25	67.16	70.28
13	-2.113	60.340	0.094	52.76	56.84	60.34	64.61	67.33	69.38	72.83	-1.606	60.376	0.098	52.28	56.69	60.38	64.77	67.49	69.51	72.82
14	-1.950	62.112	0.096	54.14	58.45	62.11	66.55	69.35	71.46	74.97	-1.599	61.951	0.099	53.59	58.14	61.95	66.49	69.31	71.41	74.84
15	-1.858	63.863	0.096	55.58	60.06	63.86	68.45	71.33	73.49	77.06	-1.591	63.060	0.099	54.56	59.19	63.06	67.67	70.53	72.65	76.13
16	-1.776	65.493	0.097	56.93	61.57	65.49	70.19	73.13	75.33	78.95	-1.583	63.801	0.098	55.24	59.90	63.80	68.43	71.31	73.43	76.92
17	-1.680	66.985	0.097	58.19	62.97	66.99	71.77	74.74	76.95	80.57	-1.576	64.322	0.098	55.71	60.40	64.32	68.97	71.85	73.99	77.48
18	-1.537	68.370	0.096	59.36	64.28	68.37	73.19	76.16	78.35	81.92	-1.568	64.660	0.098	56.01	60.72	64.66	69.33	72.22	74.36	77.86
19	-1.344	69.682	0.095	60.45	65.52	69.68	74.52	77.46	79.61	83.07	-1.561	64.891	0.098	56.20	60.93	64.89	69.58	72.48	74.63	78.14

BODY MASS INDEX (BMI) (N= 64,262)																				
Age (Years)	Males										Females									
	L	M	S	P5	P25	P50	P75	P85	P90	P95	L	M	S	P5	P25	P50	P75	P85	P90	P95
5	-11.073	13.748	0.103	12.45	12.94	13.45	14.21	14.80	15.30	16.22	-4.395	13.662	0.072	12.42	13.07	13.66	14.42	14.95	15.36	16.12
6	-10.551	14.008	0.118	12.55	13.08	13.63	14.47	15.11	15.65	16.65	-4.197	13.606	0.077	12.30	12.98	13.60	14.42	14.98	15.43	16.26
7	-10.029	14.276	0.133	12.65	13.23	13.82	14.73	15.43	16.01	17.10	-3.999	13.669	0.082	12.27	13.00	13.67	14.55	15.16	15.65	16.56
8	-9.506	14.562	0.147	12.77	13.39	14.03	15.02	15.78	16.41	17.59	-3.802	13.810	0.088	12.31	13.09	13.81	14.76	15.43	15.98	17.00
9	-8.984	14.872	0.158	12.93	13.59	14.29	15.36	16.18	16.87	18.16	-3.604	14.079	0.095	12.44	13.29	14.08	15.13	15.88	16.49	17.63
10	-8.462	15.211	0.163	13.14	13.85	14.60	15.76	16.65	17.41	18.82	-3.406	14.513	0.102	12.71	13.64	14.51	15.68	16.52	17.21	18.51
11	-7.939	15.580	0.164	13.41	14.18	14.98	16.23	17.20	18.03	19.58	-3.208	15.108	0.108	13.12	14.15	15.10	16.41	17.35	18.12	19.60
12	-7.417	15.976	0.159	13.74	14.56	15.43	16.77	17.83	18.73	20.44	-3.010	15.753	0.114	13.57	14.70	15.75	17.19	18.22	19.08	20.70
13	-6.895	16.396	0.151	14.12	15.00	15.93	17.37	18.51	19.50	21.38	-2.812	16.571	0.119	14.18	15.41	16.57	18.14	19.26	20.19	21.93
14	-6.372	16.835	0.141	14.55	15.49	16.47	18.00	19.22	20.28	22.34	-2.614	17.471	0.122	14.87	16.22	17.47	19.16	20.35	21.33	23.15
15	-5.850	17.286	0.131	15.00	16.00	17.03	18.63	19.91	21.03	23.25	-2.416	17.970	0.123	15.24	16.66	17.97	19.71	20.92	21.90	23.70
16	-5.328	17.744	0.122	15.45	16.51	17.58	19.22	20.53	21.70	24.03	-2.218	18.355	0.124	15.52	17.00	18.35	20.13	21.34	22.31	24.04
17	-4.805	18.205	0.115	15.90	17.01	18.12	19.77	21.08	22.23	24.57	-2.021	18.631	0.124	15.70	17.24	18.63	20.42	21.62	22.57	24.24
18	-4.283	18.665	0.108	16.34	17.50	18.63	20.26	21.51	22.60	24.79	-1.823	18.859	0.125	15.84	17.44	18.86	20.66	21.86	22.78	24.39
19	-3.760	19.126	0.103	16.78	17.98	19.11	20.69	21.84	22.81	24.70	-1.625	19.049	0.126	15.94	17.59	19.05	20.87	22.05	22.96	24.51

Appendix Figure 2: Smoothed waist circumference (WC) and BMI percentiles curves for males and females aged 5-19 years based on a "Reference" population in analytical sample 2 (removing ±2SD BMI z scores)

WAIST CIRCUMFERENCE

Male

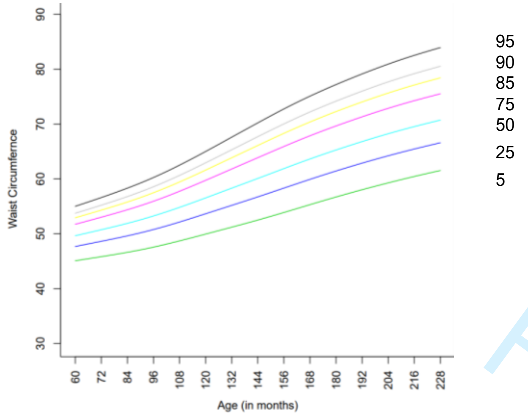

Female

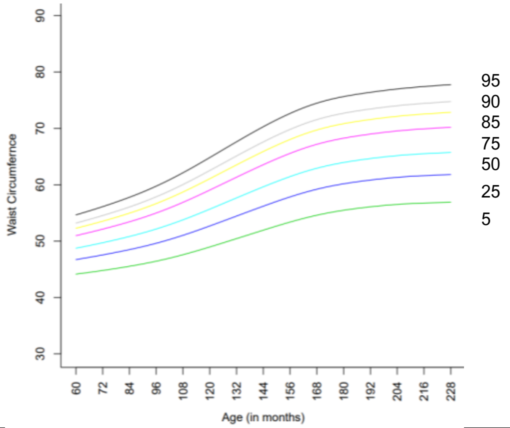

BODY MASS INDEX

Male

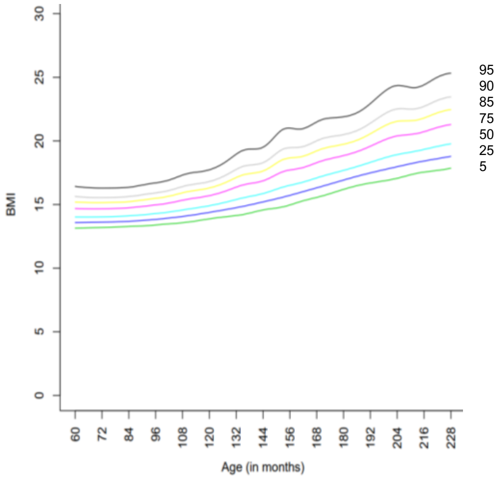

Female

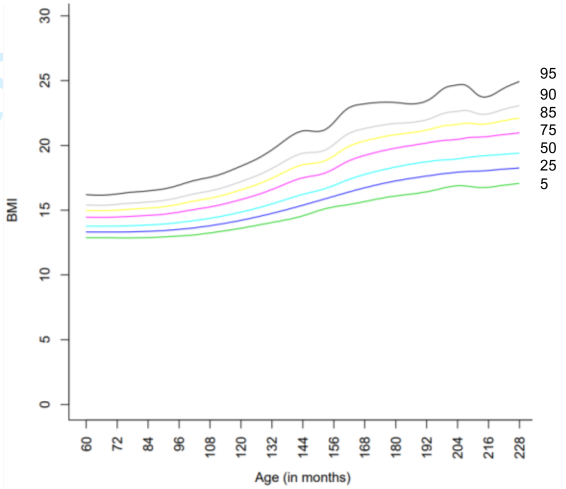

For Review

Appendix Table 3: Smoothed percentile values of WC (cm) and BMI by age and sex for males and females aged 5-19 years based on a "Reference" population in analytical sample 3 (removing ±2SD BMI z scores)

WAIST CIRCUMFERENCE (WC) (N=53,516)																				
Age (Years)	Males										Females									
	L	M	S	P5	P25	P50	P75	P85	P90	P95	L	M	S	P5	P25	P50	P75	P85	P90	P95
5	-0.629	49.641	0.060	45.08	47.68	49.64	51.73	52.92	53.74	55.01	-1.360	48.735	0.064	44.14	46.72	48.73	50.97	52.27	53.20	54.65
6	-0.680	50.743	0.064	45.83	48.63	50.74	53.02	54.31	55.22	56.61	-1.331	49.714	0.068	44.79	47.55	49.71	52.12	53.53	54.54	56.12
7	-0.731	51.907	0.068	46.63	49.62	51.91	54.38	55.79	56.79	58.32	-1.303	50.822	0.072	45.53	48.49	50.82	53.43	54.96	56.06	57.78
8	-0.782	53.248	0.072	47.57	50.78	53.25	55.94	57.48	58.58	60.26	-1.275	52.147	0.076	46.44	49.62	52.15	54.99	56.66	57.86	59.75
9	-0.833	54.819	0.076	48.70	52.15	54.82	57.75	59.45	60.65	62.51	-1.246	53.777	0.080	47.58	51.03	53.78	56.88	58.72	60.04	62.13
10	-0.884	56.545	0.080	49.94	53.65	56.54	59.75	61.61	62.94	65.01	-1.218	55.669	0.085	48.96	52.68	55.67	59.06	61.07	62.53	64.83
11	-0.935	58.315	0.084	51.21	55.18	58.32	61.81	63.86	65.32	67.61	-1.189	57.664	0.088	50.44	54.44	57.66	61.33	63.52	65.11	67.62
12	-0.986	60.092	0.088	52.50	56.73	60.09	63.87	66.10	67.70	70.22	-1.161	59.632	0.091	51.94	56.20	59.63	63.56	65.90	67.60	70.30
13	-1.037	61.886	0.091	53.88	58.33	61.89	65.92	68.31	70.03	72.76	-1.132	61.446	0.093	53.38	57.84	61.45	65.57	68.03	69.82	72.65
14	-1.088	63.637	0.092	55.31	59.93	63.64	67.86	70.38	72.20	75.09	-1.104	62.926	0.093	54.59	59.20	62.93	67.18	69.72	71.55	74.47
15	-1.139	65.281	0.093	56.71	61.45	65.28	69.66	72.28	74.18	77.20	-1.075	63.947	0.093	55.47	60.17	63.95	68.26	70.83	72.69	75.64
16	-1.190	66.812	0.093	58.03	62.88	66.81	71.33	74.04	76.01	79.15	-1.047	64.647	0.093	56.08	60.83	64.65	68.99	71.57	73.44	76.40
17	-1.241	68.239	0.093	59.28	64.22	68.24	72.87	75.66	77.69	80.94	-1.018	65.166	0.093	56.51	61.31	65.17	69.54	72.14	74.02	76.99
18	-1.292	69.544	0.093	60.45	65.46	69.54	74.27	77.12	79.20	82.53	-0.989	65.503	0.094	56.75	61.61	65.50	69.92	72.54	74.43	77.42
19	-1.343	70.734	0.093	61.54	66.60	70.73	75.53	78.43	80.55	83.95	-0.961	65.736	0.094	56.88	61.80	65.74	70.20	72.85	74.76	77.77

BODY MASS INDEX (BMI) (N= 53,516)																				
Age (Years)	Males										Females									
	L	M	S	P5	P25	P50	P75	P85	P90	P95	L	M	S	P5	P25	P50	P75	P85	P90	P95
5	-12.610	14.217	0.078	13.06	13.48	13.91	14.56	15.05	15.47	16.25	-13.108	14.109	0.101	12.87	13.31	13.77	14.46	14.97	15.41	16.20
6	-13.935	14.284	0.083	13.18	13.59	14.00	14.63	15.10	15.50	16.22	-12.900	14.128	0.103	12.86	13.31	13.78	14.48	15.00	15.45	16.25
7	-14.290	14.400	0.088	13.29	13.71	14.14	14.79	15.28	15.69	16.43	-12.183	14.208	0.106	12.89	13.36	13.85	14.59	15.16	15.63	16.49
8	-13.508	14.598	0.093	13.42	13.87	14.34	15.04	15.58	16.02	16.83	-11.227	14.396	0.108	13.00	13.52	14.05	14.85	15.47	15.98	16.94
9	-12.121	14.889	0.098	13.59	14.10	14.62	15.41	16.01	16.51	17.42	-10.421	14.717	0.110	13.24	13.80	14.37	15.25	15.92	16.49	17.54
10	-12.171	15.268	0.103	13.89	14.41	14.95	15.76	16.37	16.88	17.81	-9.499	15.174	0.111	13.60	14.22	14.85	15.82	16.57	17.21	18.40
11	-9.786	15.720	0.108	14.15	14.77	15.42	16.41	17.17	17.81	19.00	-8.122	15.746	0.110	14.05	14.75	15.48	16.60	17.47	18.22	19.66
12	-10.019	16.245	0.113	14.59	15.23	15.90	16.92	17.70	18.36	19.59	-6.765	16.405	0.108	14.57	15.38	16.22	17.50	18.51	19.39	21.12
13	-8.265	16.851	0.117	14.97	15.73	16.52	17.73	18.68	19.49	21.02	-7.168	17.110	0.106	15.25	16.07	16.91	18.19	19.19	20.07	21.78
14	-8.961	17.516	0.121	15.54	16.29	17.06	18.25	19.17	19.95	21.42	-4.312	17.787	0.103	15.66	16.73	17.76	19.23	20.34	21.30	23.21
15	-9.860	18.199	0.125	16.15	16.86	17.60	18.73	19.61	20.34	21.71	-3.465	18.340	0.100	16.10	17.26	18.34	19.79	20.83	21.69	23.31
16	-9.328	18.859	0.130	16.62	17.41	18.22	19.46	20.43	21.25	22.79	-3.050	18.739	0.099	16.42	17.63	18.74	20.19	21.18	21.99	23.44
17	-8.157	19.460	0.135	17.02	17.92	18.86	20.29	21.41	22.39	24.23	-5.089	19.016	0.099	16.88	17.93	18.96	20.47	21.63	22.64	24.67
18	-9.101	19.994	0.142	17.55	18.43	19.34	20.73	21.81	22.74	24.48	-2.578	19.223	0.100	16.77	18.07	19.22	20.69	21.67	22.44	23.78
19	-8.566	20.486	0.149	17.92	18.90	19.92	21.49	22.72	23.78	25.81	-3.796	19.407	0.101	17.06	18.26	19.40	20.96	22.11	23.07	24.94

For Review Only

Appendix Figure 3: Comparison of sex wise data distribution of WC and BMI percentiles values with 95 % confidence interval in Samples 1, 2 and 3

50th and 90th percentiles for Waist Circumference by age and sex for all eligible participants and after excluding ±2sd and ±3sd

Males **Females**

50th percentiles

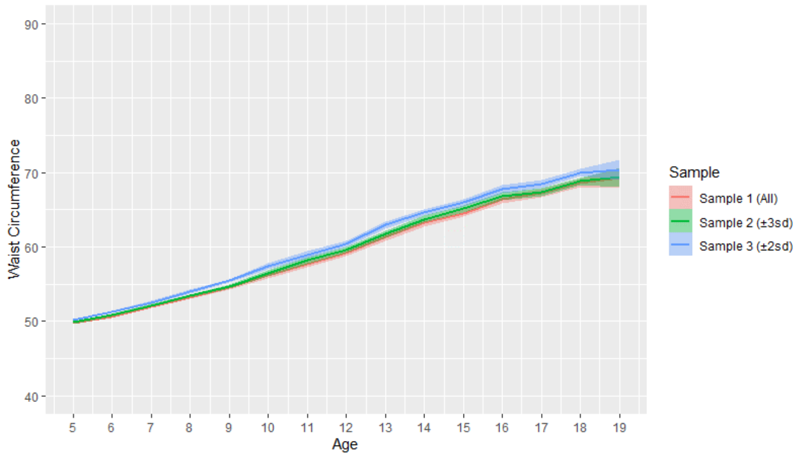

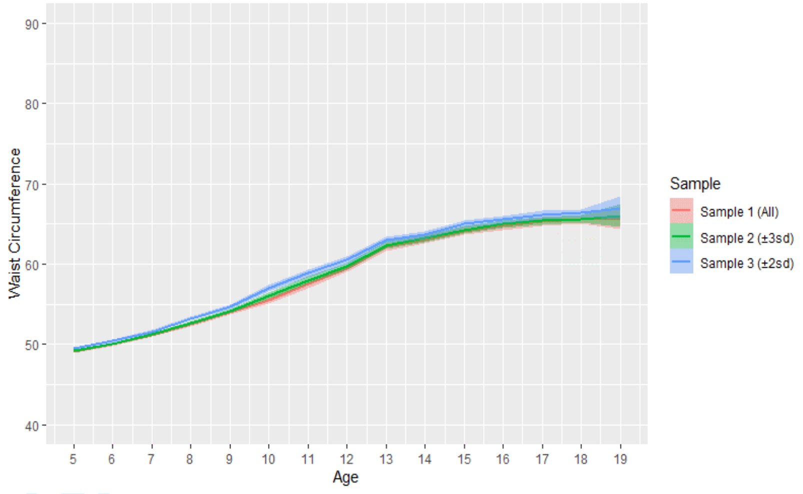

90th percentiles

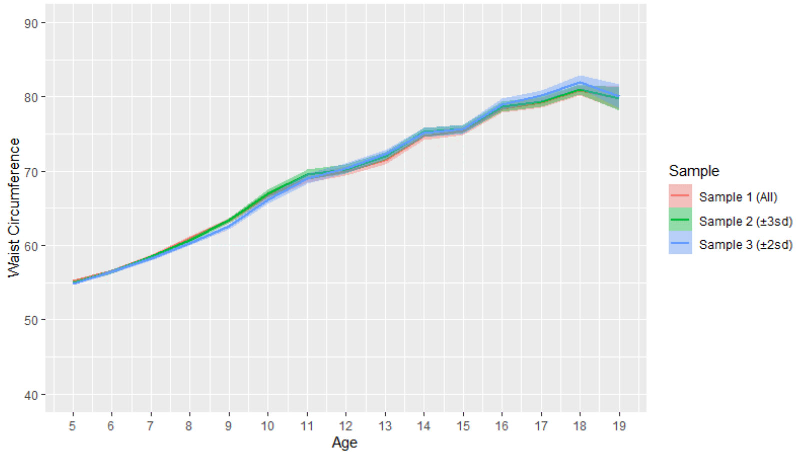

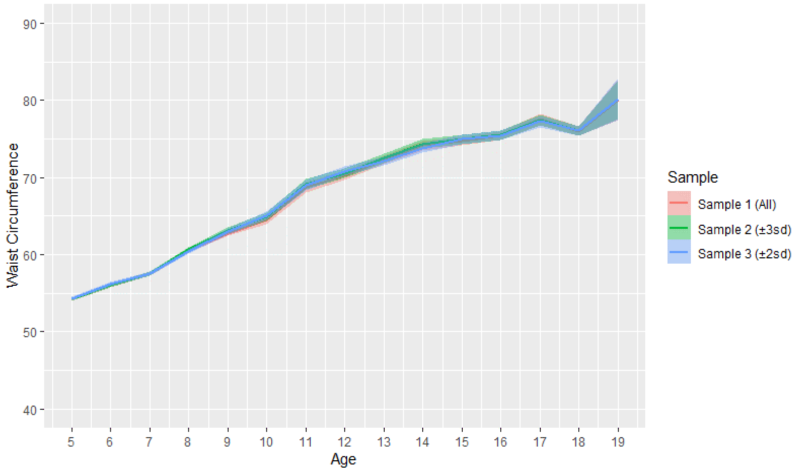

50th and 95th percentile with 95%CI for BMI by age and sex based for all eligible participants and after excluding ±2sd and ±3sd

Males **Females**

50th percentiles

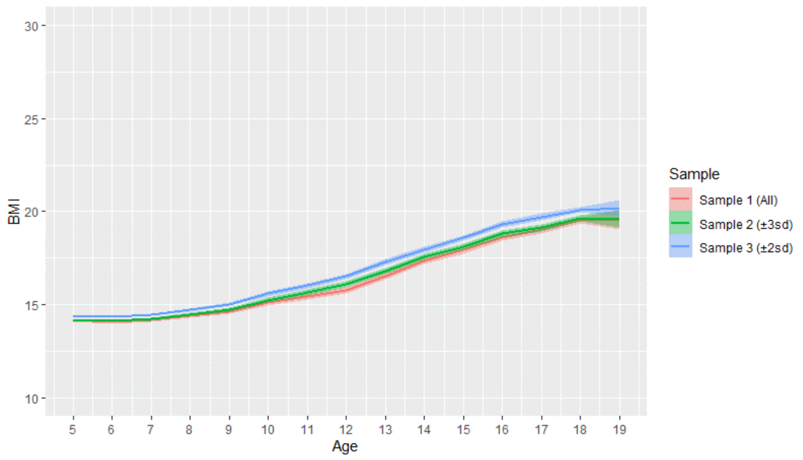

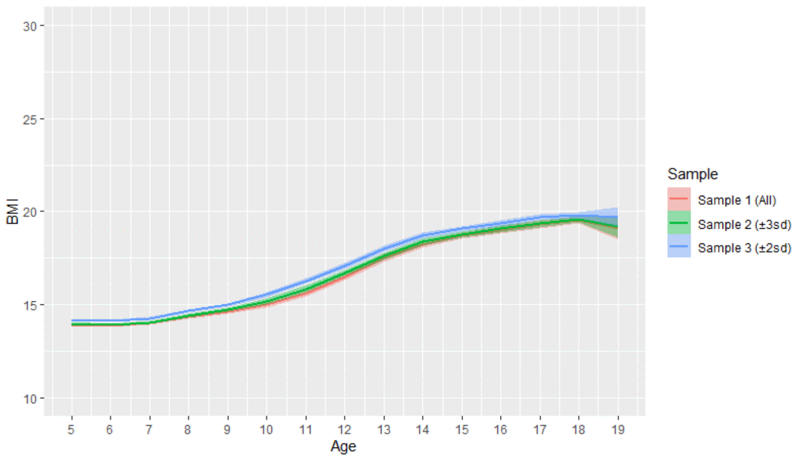

95th percentiles

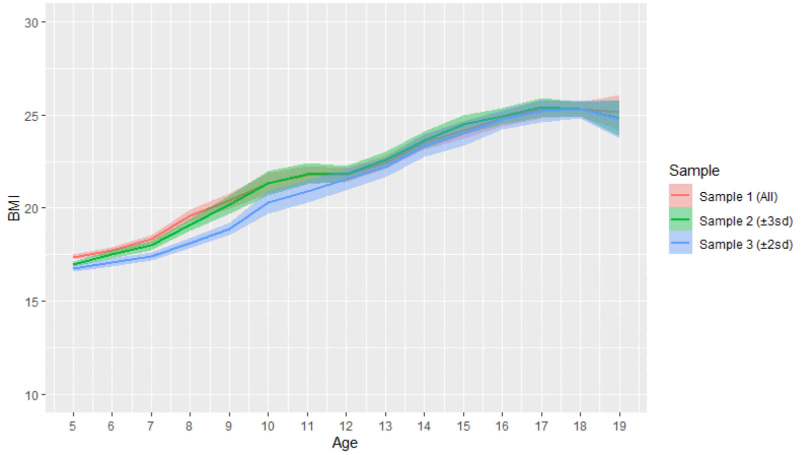

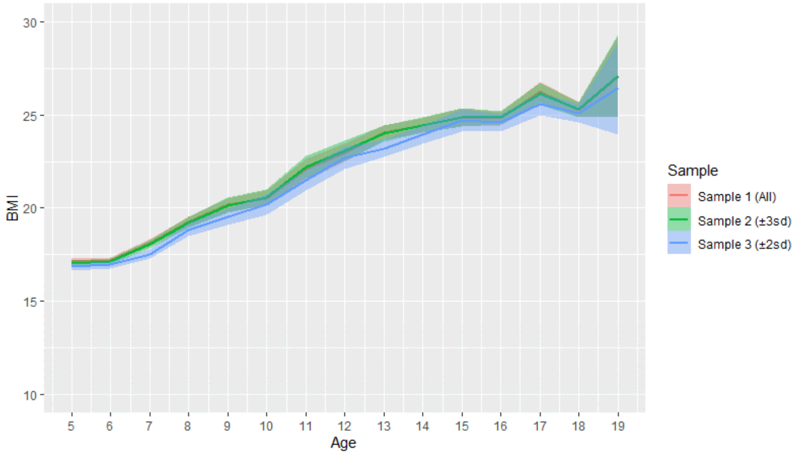

Appendix Table 4: Comparison of smoothed 50th and 90th percentiles of Waist circumference for Boys and Girls at age 10, 14 and 18 years

Age group	10 years						14 years						18 years					
Country	India[1]	US[2]	Malaysia[3]	Pakistan[4]	Turkey[5]	Poland[6]	India[1]	US[2]	Malaysia[3]	Pakistan[4]	Turkey[5]	Poland[6]	India[1]	US[2]	Malaysia[3]	Pakistan[4]	Turkey[5]	Poland[6]
Boys																		
50th	55.1	65.9	58.7	60.3	60.5	61.4	61.4	75.0	67.5	NA	68.9	69.1	67.8	84.1	NA	NA	NA	76.4
90th	62.6	83.3	74.5	72.0	69.5	72.9	71.0	99.2	86.4	NA	79.5	81.0	78.5	115.1	NA	NA	NA	86.5
Girls																		
50th	54.4	66.1	56.7	61.0	58.2	59.1	61.5	75.4	65.6	NA	64.7	66.2	64.6	84.7	NA	NA	NA	69.0
90th	61.9	84.5	69.8	74.0	66.3	69.5	71.2	100.5	80.1	NA	73.3	77.9	74.4	116.5	NA	NA	NA	78.2

Study year: [1]2016-2018; [2]2009-2014; [3]2008-2009; [4]2009-2010; [5]2005; [6] 2002-2005
NA-Not available

Appendix Table 5: Comparison of smoothed 50th and 90th percentiles of Body mass index for Boys and Girls at age 10, 14 and 18 years

Age group	10 years						14 years						18 years					
Country	India[1]	US[2]	Malaysia[3]	Pakistan[4]	Turkey[5]	Poland[6]	India[1]	US[2]	Malaysia[3]	Pakistan[4]	Turkey[5]	Poland[6]	India[1]	US[2]	Malaysia[3]	Pakistan[4]	Turkey[5]	Poland[6]
Boys																		
50th	14.5	16.4	16.6	14.5	17.7	17.1	16.3	19.0	18.6	NA	19.7	19.2	18.7	21.7	NA	NA	21.7	21.8
90th	17.1	19.2	23.2	19.5	21.1	21.7	19.9	22.8	25.6	NA	23.7	24.1	22.7	26.0	NA	NA	25.8	26.4
Girls																		
50th	14.5	16.6	16.3	16.2	17.5	16.9	17.2	19.6	18.4	NA	20.2	19.4	18.9	21.3	NA	NA	21.6	20.7
90th	17.3	19.9	22.0	21.4	20.9	21.2	21.2	23.8	23.6	NA	25.6	24.1	22.9	26.0	NA	NA	25.2	24.9

Study year: [1]2016-2018; [2]2007; [3]2011; [4]2009-2010; [5]2005; [6] 2007-2009
NA-Not available

For Review Only
